# Computerized clinical decision support systems for primary preventive care: A decision-maker-researcher partnership systematic review of effects on process of care and patient outcomes

**DOI:** 10.1186/1748-5908-6-87

**Published:** 2011-08-03

**Authors:** Nathan M Souza, Rolf J Sebaldt, Jean A Mackay, Jeanette C Prorok, Lorraine Weise-Kelly, Tamara Navarro, Nancy L Wilczynski, R Brian Haynes

**Affiliations:** 1 Health Research Methodology Program, McMaster University, 1280 Main Street West, Hamilton, ON, Canada; 2 Department of Medicine, McMaster University, 1280 Main Street West, Hamilton, ON, Canada; 3 Health Information Research Unit, Department of Clinical Epidemiology and Biostatistics, McMaster University, 1280 Main Street West, Hamilton, N, Canada; 4 Hamilton Health Sciences, 1200 Main Street West, Hamilton, ON, Canada

## Abstract

**Background:**

Computerized clinical decision support systems (CCDSSs) are claimed to improve processes and outcomes of primary preventive care (PPC), but their effects, safety, and acceptance must be confirmed. We updated our previous systematic reviews of CCDSSs and integrated a knowledge translation approach in the process. The objective was to review randomized controlled trials (RCTs) assessing the effects of CCDSSs for PPC on process of care, patient outcomes, harms, and costs.

**Methods:**

We conducted a decision-maker-researcher partnership systematic review. We searched MEDLINE, EMBASE, Ovid's EBM Reviews Database, Inspec, and other databases, as well as reference lists through January 2010. We contacted authors to confirm data or provide additional information. We included RCTs that assessed the effect of a CCDSS for PPC on process of care and patient outcomes compared to care provided without a CCDSS. A study was considered to have a positive effect (*i.e*., CCDSS showed improvement) if at least 50% of the relevant study outcomes were statistically significantly positive.

**Results:**

We added 17 new RCTs to our 2005 review for a total of 41 studies. RCT quality improved over time. CCDSSs improved process of care in 25 of 40 (63%) RCTs. Cumulative scientifically strong evidence supports the effectiveness of CCDSSs for screening and management of dyslipidaemia in primary care. There is mixed evidence for effectiveness in screening for cancer and mental health conditions, multiple preventive care activities, vaccination, and other preventive care interventions. Fourteen (34%) trials assessed patient outcomes, and four (29%) reported improvements with the CCDSS. Most trials were not powered to evaluate patient-important outcomes. CCDSS costs and adverse events were reported in only six (15%) and two (5%) trials, respectively. Information on study duration was often missing, limiting our ability to assess sustainability of CCDSS effects.

**Conclusions:**

Evidence supports the effectiveness of CCDSSs for screening and treatment of dyslipidaemia in primary care with less consistent evidence for CCDSSs used in screening for cancer and mental health-related conditions, vaccinations, and other preventive care. CCDSS effects on patient outcomes, safety, costs of care, and provider satisfaction remain poorly supported.

## Background

Achieving comprehensive and effective primary preventive care (PPC) remains a challenge for healthcare systems worldwide. Despite the existence of clinical guidelines, many preventive care interventions are still underused, for example, the low influenza vaccine rates among children and adolescents with increased-risk conditions [1] and the limited use of prophylaxis against deep vein thrombosis [2].

Interventions to overcome this problem may affect healthcare governance, financial, and delivery arrangements, and may include use of health information technologies such as electronic health records and computerized clinical decision support systems (CCDSSs). CCDSSs have been promoted in many high-income countries as a promising tool for improving PPC [3]. The USA and other nations have accelerated their implementation as part of stimulus packages issued in 2009 [4,5].

We define CCDSSs for PPC as computerized matching of an individual patient's characteristics with a knowledge base that then provides patient-specific recommendations to healthcare providers about PPC. Despite their promise and expense, definitive evidence of CCDSS effectiveness for process of care (*e.g*., performance and satisfaction of healthcare providers), patient outcomes (*e.g*., functional status, disability, major clinical events, quality of life, and death), costs, and safety remain to be established [6-8].

Our previous review showed inconsistent evidence of improvement in providers' adherence to PPC procedures such as screening for breast, cervical, and prostate cancers, and very weak evidence on improvement of patient outcomes [6]. Another review found modest effectiveness for CCDSSs that prompt clinicians for smoking cessation interventions (average increase in delivery of preventive care measure: 23%), cardiac care (average increase: 20%), blood pressure screening (average increase: 16%), vaccinations, diabetes management, and cholesterol (average increase for each measure: 15%), and mammographic screening (average increase: 10%), but only eight (13%) of the included studies tested fully computerized reminders [9]. Jacobson and Szilagyi showed that patient reminder and recall systems in primary care settings are effective in improving immunization rates in developed countries [10]. However, effects of CCDSSs on patient outcomes, costs, and safety have yet to be shown [11,12].

Many new studies have been published recently, and many health care institutions and clinical practices are considering implementation of this new information technology. We conducted a systematic review of randomized controlled trials (RCTs) assessing the effectiveness of CCDSSs for PPC on process of care, patient outcomes, costs, safety, and provider satisfaction with CCDSS for PPC in partnership with clinical decision makers.

## Methods

The detailed methods for this systematic review have been published elsewhere [13] and are available through open access http://www.implementationscience.com/content/5/1/12.

### Research questions

This systematic review addressed two questions: Do CCDSSs improve process of care or patient outcomes for PPC, and what are the costs, safety, and provider satisfaction with CCDSS for PPC?

### Partnering with decision makers

The review team included a partnership between McMaster University's Health Information Research Unit (HIRU), the senior administration of Hamilton Health Sciences (a large Canadian academic health sciences centre) and Local Health Integration Network (the regional health authority that includes Hamilton), and clinical service chiefs at local hospitals. Decision-maker partners were included in discussions about data extraction for, and interpretation of, factors that might affect implementation. The decision-maker-researcher partnership hypothesized positive effects of CCDSSs in both process of care and patient outcomes regarding PPC, methodological improvement in testing of CCDSSs over time, cost savings, and improved safety and provider satisfaction with CCDSS use.

### Search strategy

We previously described our search methods up to 2004 [6] and for this update [13]. Briefly, for the latest update we used a comprehensive search strategy to retrieve potentially relevant RCTs from MEDLINE, EMBASE, Ovid's Evidence-Based Medicine Reviews, and the Inspec bibliographic database from 1 January 2004 to 8 December 2008; a further update was conducted to 6 January 2010. We performed duplicate screening of eligible RCTs and independent data-extraction using piloted forms that were constructed with our decision-maker partners; a third reviewer resolved disagreements. Inter-reviewer agreement on study eligibility was measured using the unweighted Cohen's kappa (κ), and was excellent (κ = 0.93; 95% confidence interval [CI], 0.91 to 0.94) over all applications. Study authors confirmed extracted data for 88% (36/41) of the studies included in the PPC review.

### Study selection

We included RCTs (including cluster RCTs) published in any language that compared the effects of care with a CCDSS for PPC, used by healthcare providers, with care without a CCDSS. Outcomes included processes of care and patient outcomes. We only considered RCTs because this method minimizes the risk of biased allocation, and there has been increased publication of RCTs since our 2005 review [6].

For PPC interventions, patients had to be free from the illness to be prevented (*e.g*., a specific strain of influenza) but could be seen in any setting, including acute healthcare. CCDSSs that provided only computer-aided instruction, performed actions unrelated to clinical decision making (*e.g*., CCDSSs for diagnostic performance against a gold standard), or evaluated CCDSS users' knowledge or performance in clinical simulations were excluded.

We excluded studies where PPC interventions were merged with a complex set of other interventions (*e.g*., chronic disease management) and those that did not focus on PPC (*e.g*., screening of medical errors). We did however include one study that evaluated a CCDSS for influenza vaccination in asthmatic patients because it provided evidence about the independent effects of the intervention on vaccination rates [1].

### Data extraction

Independent reviewers extracted key data in duplicate, including study methods, CCDSS and population characteristics, possible sources of bias, and outcomes. Primary authors of each study were asked to review the extracted data for their study and offer comments on the extracted data.

### Assessment of study quality

Details of our quality assessment of included RCTs are published elsewhere [13]. RCTs were scored for methodological quality on a 10-point scale (an extension of the Jadad scale [14]) with scores ranging from 0 for the lowest study quality to 10 for the highest quality.

### Assessment of CCDSS intervention effects

Researchers and decision-makers selected outcomes that were relevant to PPC from each study before evaluating intervention effects. We used RCTs as the unit of analysis to assess CCDSS effectiveness. A process of care outcome represents the delivered quality of care, while a patient outcome represents the directly measured health status of the patient. We used a dichotomous measure of effect and defined a CCDSS as effective (positive) when there was a significant (*p*< 0.05) improvement in the endpoint specified as main or primary by the authors or, if no primary endpoint was specified, the endpoint used to estimate study power, or, failing that, ≥50% of multiple pre-specified endpoints. When no clear pre-specified endpoints existed, we considered a CCDSS effective if it improved ≥50% of all reported outcomes. Studies that included ≥1 CCDSS treatment arm were considered effective if any of the treatment CCDSS arms was evaluated as effective. These criteria are more specific than in our 2005 review [6], and the effect assignment was adjusted for some studies from that review.

### Data synthesis and analysis

We used descriptive summary measures for data including proportions for categorical variables and means (± standard deviations) for continuous variables. When reporting results from individual studies, we cited the measures of association and *p*-values as reported in the studies. We considered methodological rigor and scientific quality of the included trials to analyze data and formulate conclusions. We did not pool data or compare studies using effect sizes because of study heterogeneity in populations, settings, interventions, and outcomes. A sensitivity analysis was conducted to assess the possibility of biased results in studies with a mismatch between the unit of allocation (*e.g*., clinicians) and the unit of analysis (*e.g*., individual patients without adjustment for clustering). Success rates comparing studies with matched and mismatched analyses were compared using chi-square for comparisons. No differences in reported success were found for either process of care outcomes (Fisher's exact test, 2*P *= 1.0) or patient outcomes (Fisher's exact test, 2*P *= 1.0). Accordingly, results have been reported without distinction for mismatch.

## Results

**Figure 1:**
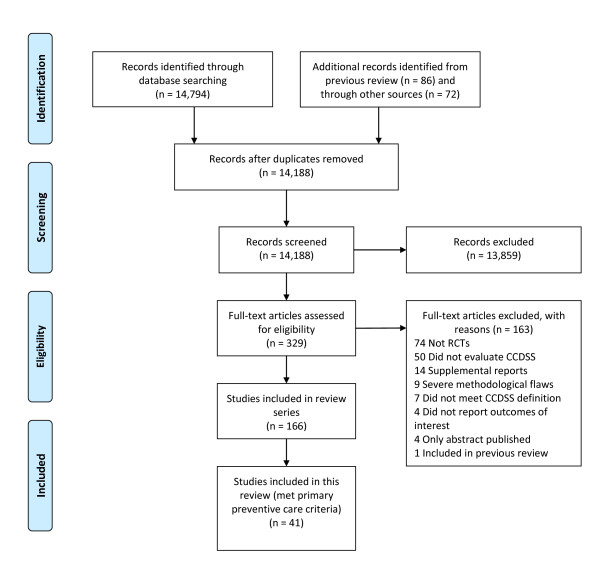
Flow diagram of included and excluded studies for the update 1 January 2004 to 6 January 2010 with specifics for primary preventive care*. *Details provided in: Haynes RB *et al. *[13]. Two updating searches were performed, for 2004 to 2009 and to 6 January 2010 and the results of the search process are consolidated here.

We included 46 publications describing 41 trials (Figure 1) [1,15-59]. We excluded five of the 24 studies included in our previous review [6] because they did not meet our new, stricter inclusion criteria [60-63] or were more relevant for another application [64]. Additionally, we excluded 14 RCTs because reminders were part of a more complex intervention for chronic disease including diabetes [65-69], hypertension [70,71], heart failure and/or ischemic heart disease [72], asthma or chronic obstructive pulmonary disease [73], or the CCDSS screened for medical errors [74,75] including those caused by drug-drug interaction and adverse drug effects [76], reported on advanced clinical directives [77], or compared two CCDSSs [78]. Twelve included studies contribute outcomes to this review as well as other CCDSS applications in the series; two studies [27,28] to four reviews, five studies [18,19,29,31,42,59] to three reviews, and five studies [1,43-45,47,50,56] to two reviews; but we focused here on PPC-relevant outcomes.

Summary outcome data are reported in Table 1. The methodological quality of included studies is summarized in Additional file 1 Table S1; CCDSS characteristics in Additional file 2 Table S2; study characteristics in Additional file 3 Table S3; detailed outcome data in Additional file 4 Table S4; and other CCDSS process-related outcomes in Additional file 5 Table S5.

**Table 1:** Summary of results of CCDSS trials of primary preventive care

Study	Method Score	Indication	No. of centres/providers/patients	Process of care outcomes	CCDSS Effect^a^	Patient outcomes	CCDSS Effect^a^
**Cancer screening**							

Sequist, 2009 [49]	9	Reminders to screen for colorectal cancer in primary care.	11 / 110* / 21,860	Individual tests performed: FOBT; Flexible sigmoidoscopy; Colonoscopy.	**0**	Pathologic findings: Colonic adenoma; Colorectal cancer.	**0**
Emery, 2007 [30]	10	Recommendations for assessment and management of familial cancer risk in primary care.	45* /.../ 219	Appropriate referrals to regional genetics clinic.	**+**	Cancer worry score; Risk perception score; Accuracy of patient risk perception; Knowledge about familial cancer.	**0**
Wilson, 2005 [57,58]	6	Recommendations for referral and provision of information for breast cancer genetic risk in primary care.	86* / 243 / 242	Confidence in management of patients with family history of breast cancer concerns.	**0**	Perception of risk; Understanding of 'incorrect' breast cancer risk factors.	**0**
Burack, 2003 [24]	8	Reminders for mammography and pap smear tests in primary care.	3 / 20 / 2,471*	Primary care visit during study year; Mammogram completed during study year; Pap smear test completed during study year.	**0**	...	**..**.
Burack, 1998 [23]	6	Reminders to perform pap smear screening in primary care.	3 / 20 / 5,801*	Patients with primary care visit; Patients with pap smear completed.	**0 **	...	**..**.
Burack, 1997 [22]	8	Reminders for mammography in primary care.	3 / 25 / 2,826*	Mammography completion rates.	**+**	...	**..**.
Burack, 1996 [21]	8	Reminders for mammography screening in primary care.	2 / 20 / 2,368*	Primary care visit for women due for mammography; Mammography rates.	**0**	...	**..**.
Burack, 1994 [20]	8	Reminders for mammography in primary care.	5 / 25 / 2,725*	Proportion of women with scheduled mammography appointments; Proportion of women having mammography.	**+**	...	**..**.
McPhee, 1991 [40]	7	Reminders for cancer screening and preventive counselling in primary care.	.../ 40* /...	Compliance with American Cancer Society and/or National Cancer Institute recommendations.	**+**	...	**..**.
McPhee, 1989 [39]	7	Reminders for cancer screening and preventive counselling in primary care.	1 / 62* / 1,936	Compliance with recommendations for FOBT, rectal exam, sigmoidoscopy, pap smear test, pelvic exam, breast exam, and mammography.	**+**	...	**..**.

**Multiple preventive care activities**							

Harari, 2008 [34]	7	Recommendations for primary preventative care and screening for functionally independent elderly patients in primary care.	4 / 26 / 2,503*	BP check, FOBT (<80 years of age), influenza vaccination, dental check, vision check-up, or hearing check-up in previous year; Cholesterol measurement in previous five years (<75 years of age); Blood glucose measurement in previous three years; Pneumococcal vaccination (ever); Mammography in previous two years (<70 years of age).	**0**	Moderate or strenuous physical activity; Consumption of high fat food items; Consumption of fruit/fibre items; No current tobacco use; No or moderate alcohol use; Driving with use of seat belt.	**0**
Apkon, 2005 [16]	5	Screening, preventive care, and recommendations for management of acute or chronic conditions for ambulatory care patients in military facilities.	3 / 12 / 1,902*	Screening/prevention healthcare opportunities fulfilled; Acute/chronic healthcare opportunities (lipid abnormalities); Patient satisfaction.	**0**	Adverse events.	**0**
Dexter, 2001 [29]	10	Reminders for preventive therapies in hospital inpatients.	...* / 202 / 3,416	Proportion of hospitalizations with an order for therapy (all patients and only eligible patients).	**+**	...	**..**.
Demakis, 2000 [28]	7	Reminders for screening, monitoring, and counselling in accordance with predefined standards of care in ambulatory care.	12* / 275 / 12,989	Per-patient and per-visit compliance with standards of care related to hypertension (weight, exercise, sodium), nutrition counselling for diabetes, and pneumococcal vaccination for elderly or high-risk patients.	**+**	...	**..**.
Overhage, 1996 [42]	10	Reminders to comply with 22 US Preventive Services Task Force preventive care measures for hospital inpatients.	1* / 78 / 1,622	Compliance with preventive care guidelines; Attitude towards providing preventive care to hospitalised patients.	**0**	...	**..**.
Frame, 1994 [33]	6	Reminders for cancer screening, CV disease preventive screening, identification of at-risk behavior, patient education, and vaccination in primary care.	5 / 12 / 1,324*	Change in provider compliance with 11 health maintenance procedures over two years.	**+**	...	**..**.
Turner, 1994 [53]	5	Reminders for cancer screening and influenza vaccination in primary care.	44* / 44 / 740	Performance of health maintenance activities including influenza vaccinations, FOBTs, pap smears, breast exams, and mammography.	**0**	...	**..**.
Ornstein, 1991 [41]	7	Reminders for preventive care services for adults in family medicine clinic.	1* / 49 / 7,397	Proportion of patients who received each of five preventive services.	**+ for combined reminders** **0 for physician or patient reminders**	...	**..**.
Rosser, 1991 [46]	6	Reminders for cancer screening, BP measurement, assessment of smoking status, and vaccination in outpatients.	1 /.../ 5,883*	Percentage of patients for whom the recommended procedures were performed.	**+**	...	**..**.
Tierney, 1986 [52]	6	Reminders of preventive care protocols for outpatients.	1* / 135 / 6,045	Physician compliance with preventive care protocols for fecal blood testing, pneumococcal vaccination, antacids, tuberculosis skin testing, calcium supplements, cervical cytology, mammography, and saclicylates.	**+**	...	**..**.

**Screening and management of CV risk factors**							

Bertoni, 2009 [18,19]	9	Recommendations for screening and treatment of dyslipidaemia in primary care.	59* / ... / 3,821	Patients with appropriate lipid management at follow-up.	**+**	...	**..**.
Van Wyk, 2008 [56]	10	On-demand and automatic alerts to screen and treat dyslipidaemia in primary care.	38* / 80 / 92,054	Screening of appropriate patients.	**Auto, +** **On-demand, 0**	...	**..**.
Unrod, 2007 [54,55]	8	Recommendations to increase smoking cessation counselling and quit rates in primary care.	... / 70* / 465	Physician implementation of guideline including assessment and discussion of smoking behavior, support interventions for quitting, and referral to quit-smoking programs.	**+**	Seven-day point-prevalence for abstinence.	**0**
Cobos, 2005 [27]	10	Recommendations for treatment, monitoring and follow-up for patients with dyslipidaemia in primary care.	42* /.../ 2,221	Treatment with lipid-lowering drugs in patients without coronary heart disease.	**+**	Successful management of patients without coronary heart disease.	**0**
Kenealy, 2005 [35]	10	Reminders for screening for diabetes in outpatients.	66* / 107 / 5,628	Percentage of eligible patients visiting a practitioner and screened for diabetes.	**+**	...	**..**.
Filippi, 2003 [31]	7	Reminders to prescribe acetylsalicylic acid or other antiplatelet agents to diabetic primary care patients.	... / 300* / 15,343	Antiplatelet drug prescription for patients with cardiac risk factors but without CVD.	**+**	...	**..**.
Lowensteyn, 1998 [38]	6	Calculation of coronary risk factor profile for outpatients and identification of high-risk patients in primary care.	24* / 253 / 958	Ratio for high-risk/low-risk patients returning for reassessment at three months.	**+**	Total cholesterol; Total / high-density lipoprotein cholesterol ratio; Body mass index; High-density lipoprotein cholesterol; Low-density lipoprotein cholesterol; Systolic BP; Diastolic BP; Proportion of smokers; eight-year coronary risk; CV age.	**+**
Rogers, 1984 [43-45]	4	Detection of deficiencies in care and recommendations for the management of hypertension, obesity and renal disease in outpatients.	1 / ... / 484*	Number of diets given or reviewed for obesity patients; Perceived quality of communication.	**+**	Perceived health status.	**+**
Barnett, 1983 [17]	4	Reminders to follow-up patients with newly-identified elevated BP in an acute care setting.	1 / ... / 115*	Patient follow-up attempted or achieved; Repeat BP measurement recorded.	**+**	Degree of BP control.	**+**

**Screening and management of mental health-related conditions**							

Ahmad, 2009 [15]	8	Computer-assisted screening for intimate partner violence in primary care.	1 / 11 / 314*	Opportunity to discuss possibility of risk for intimate partner violence; Detection of intimate partner violence when patient identified risk as being present and recent.	**+**	...	**..**.
Thomas, 2004 [51]	7	Identification and recommendations for management of anxiety and depression in outpatients.	5 / ... / 762*	Patient satisfaction with general practitioner.	**0**	General Health Questionnaire score.	**+**
Schriger, 2001 [48]	8	Provided computerized psychiatric interview and recommendations for patient diagnosis in the emergency department.	1 / 104 / 259*	Proportion of patients assigned a psychiatric diagnosis by CCDSS who received a psychiatric diagnosis, consultation or referral in the emergency department.	**0**	...	**..**.
Cannon, 2000 [26]	4	Reminders for screening and diagnosis of mood disorder in an outpatient mental health clinic.	1 / 4 / 78*	Proportion of patients screened for mood disorder; Proportion of major depressive disorder cases with fully documented diagnostic criteria (*Diagnostic and Statistical Manual for Mental Disorders, 4^th ^edition*).	**+**	...	**..**.
Lewis, 1996 [37]	6	Provided assessment for common mental disorders in primary care.	1 / 8 / 681*	Consultations; Referrals to other professionals; Drug prescriptions.	**0**	Difference in General Health Questionnaire score.	**0**
Rubenstein, 1995 [47]	7	Computer-generated feedback designed to identify and suggest management for functional deficits in primary care.	2* / 73 / 557	Clinical problems in medical records; Patients identified as having physical, psychological or social function impairments; Functional status interventions overall and for patients with functional status problems; Physician attitudes toward managing functional status.	**..**.	Patient functional status.	**0**

**Vaccinations**							

Fiks, 2009 [1]	8	Alerts for influenza vaccination for children and adolescents with asthma in primary care.	20* / ... / 11,919	Captured opportunities for vaccination and up-to-date vaccination rates (adjusted analysis).	**0**	...	**..**.
Flanagan, 1999 [32]	3	Online reminders for tetanus, hepatitis, pneumococcal, measles, and influenza vaccinations for adults in primary care.	... / 233* / 817	Correct vaccine decisions.	**0**	...	**..**.
Chambers, 1991 [25]	6	Reminders for influenza vaccination in primary care.	1 / 30* / 686	Influenza vaccines given.	**+ for always reminders** **0 for sometimes reminders**	...	**..**.

**Other preventive care activities**							

Sundaram, 2009 [50]	7	Reminders for risk assessment and screening for HIV in primary care.	5 / 32* / 26,042	Change in HIV testing rates.	**0**	...	**..**.
Lafata, 2007 [36]	9	Reminders for osteoporosis screening for elderly, female outpatients in primary care.	15* / 123 / 10,354	Bone mineral density testing.	**+**	...	**..**.
Zanetti, 2003 [59]	8	Alert to redose prophylactic antibiotics during prolonged cardiac surgery.	1 / ... / 447*	Intraoperative redose of antibiotics.	**+**	Surgical-site infection.	**0**

Abbreviations: BP, blood pressure; CCDSS, computerized clinical decision support system; CV(D), cardiovascular disease; FOBT, fecal occult blood test; HIV, human immunodeficiency virus.

*Unit of allocation.

^a^Outcomes are evaluated for effect as positive (+) or negative (-) for CCDSS, or no effect (0), based on the following hierarchy. An effect is defined as ≥50% of relevant outcomes showing a statistically significant difference (2*p*<0.05):

1. If a single primary outcome is reported, *in which all components are applicable*, this is the only outcome evaluated.

2. If >1 primary outcome is reported, the ≥50% rule applies and only the primary outcomes are evaluated.

3. If no primary outcomes are reported (or only some of the primary outcome components are relevant) but overall analyses are provided, the overall analyses are evaluated as primary outcomes. Subgroup analyses are not considered.

4. If no primary outcomes or overall analyses are reported, or only some components of the primary outcome are relevant for the application, any reported prespecified outcomes are evaluated.

5. If no clearly prespecified outcomes are reported, any available outcomes are considered.

6. If statistical comparisons are not reported, 'effect' is designated as not evaluated (...).

## Study quality

Additional file 1 Table S1 shows an overall increase of methodological quality of RCTs over time, although this could be due, in part, to improved reporting. Eighteen of 41 (44%) studies [1,15,18-22,24,27,29,30,35,36,42,48,49,54-56,59] scored at least 8 of 10 points (*i.e*., high quality) including six trials with perfect scores [27,29,30,35,42,56]. The main methodological limitations in low-score trials were lack of allocation concealment and cluster randomization, and incomplete follow-up. The correlation of study methodological quality with CCDSSs effects on process of care was non-significant (Pearson 0.142, 95% CI -0.18 to 0.43). The same analysis could not be undertaken for patient outcomes due to the small number of studies that evaluated patients outcomes (n = 14) and that showed a positive effect (n = 4).

### CCDSS and study characteristics

Additional file 2 Table S2 shows that 20/41 (49%) CCDSSs were integrated with an electronic medical record [1,17,25,27,29,31,32,34-36,39,41-46,49,50,52,56,59] including at least five also integrated with a computerized order entry system [1,32,42,49,56] and 21/41 (51%) were stand-alone computer systems [15,16,18-22,24,26,28,30,33,37,38,40,47,48,51,53-55,57,58]. The data entry method varied across systems, with a non-practitioner decision-maker entering data on 29/39 (74%) studies [1,15,17,21,23-25,27,29,31,32,34-55,59] and automatic entry through electronic health records in 15/39 (38%) cases [1,17,27,29,31,34-36,41,42,46,49,50,56,59]. In all but one study [26], physicians used all PPC CCDSSs, either solely or shared with other healthcare providers including trainees [1,25,28,29,39,41,42,46-48,52], advanced practice nurses [1,17-19,30,50,59], physician assistants [18,19,33], and social workers [26]. No single study completely described the CCDSSs interface. Delivery methods for CCDSS recommendations varied: 17/40 studies (43%) reported use of a desktop or laptop computer [1,26-32,34-36,42,49,50,56-59]; 10/40 (25%) used existing non-prescribing staff [17,28,33,40,41,43-46,52,53,59]; 8/40 (20%) used research project staff [15,20-22,24,38,39,47]; and the remaining studies used other methods, including personal digital assistants [18,19] and paper reports [50]. CCDSSs were pilot tested in 15/33 studies (45%), providers received training on the CCDSS in 23/35 trials (66%), and the CCDSS provided suggestions at the time of care in 36/41 studies (88%). Investigators also developed the CCDSS in 28/35 studies (80%).

Twenty-nine of 41 trials (71%) were conducted in the USA [1,16-26,28,29,32,33,36,39-45,47-50,52-55,59], 5/41 (12%) in the UK [30,34,37,51,57,58], 3/41 (7%) in Canada [15,38,46], and 1/41 (2%) each in Italy [31], New Zealand [35], Spain [27], and The Netherlands [56]. Forty-four percent (18/41) of trials were published after the year 2001 including 14/41 (34%) published after the year 2005. Eighty percent (33/41) of trials reported a public funding source [1,15-24,28-30,33-35,37,39-47,49-59], 7% (3/41) a private source [27,36,48], 2% (1/41) both public and private [38], and 10% (4/41) did not report these data [25,26,31,32]. Twenty-two trials (54%) took place mainly in primary care settings [1,18-20,22,23,27,30,31,33-38,40,49-51,53-58] while 19 trials (46%) were undertaken in a combination of hospitals, specialist clinics, and primary care, or in academic centres [15-17,21,22,24-26,28,29,32,36,39,41-48,52,59]. In all but one [1] of the 41 trials, the patients were adults or elderly.

Many CCDSS interventions for PPC were tested in the included studies. Twenty-two (54%) studies evaluated multifaceted interventions with ≥3 preventive care components [15,18-23,28,30,34,35,37,39-41,46,47,49-51,53-55,57,58], including educational sessions on preventive interventions and the CCDSS, supply of materials to clinicians and/or to patients, assessments of patient and clinician attitude towards health conditions and/or the CCDSS, audit and feedback of clinician performance, academic detailing, telephone reminder to patients, elimination of out-of-pocket expenses to patients, and use of local clinician leaders. Eleven (27%) trials assessed two components [1,16,24,27,31,33,36,38,42,48,52], and the remaining eight (21%) assessed the effectiveness of a CCDSS with one component, typically a reminder (*e.g*., printed, audio, or visual) [17,25,26,29,32,43-45,56,59].

### CCDSSs effectiveness

Table 1 (see Additional file 4 Table S4 for detailed information) shows that all trials assessed the effects of CCDSSs on processes of care. Twenty-five of 40 (61%) studies showed an improved process of care using our dichotomous measure; three of those studies also included CCDSS treatment arms that did not improve process of care [26,41,56]. Four of 14 (29%) studies showed improved patient outcomes. Only 13 (32%) studies reported both process of care and patient outcomes.

### Cancer screening (10 trials)

CCDSSs improved the screening or referral of patients with breast, cervical, ovarian, colorectal, and prostate cancers in 5/10 (50%) trials [20,22,30,39,40]. Emery *et al. *[30] showed improved rate of appropriate referrals to regional genetics clinics by primary care clinicians regarding familial cancers (*i.e*., breast, ovarian, and colorectal cancers). Conversely, Burack *et al*. demonstrated no effects for reminders for mammography screening [21] and screening mammography and pap smears tests in primary care [24]. Only three studies assessed patient outcomes, and none demonstrated effects [30,49,57,58].

### Multiple preventive care activities (10 trials)

In rural and urban primary care settings and hospitals, clinicians received CCDSS recommendations for various interventions in adult and geriatric patients including cancer screening, cardiovascular (CV) risk assessment, vaccination, tuberculosis skin tests, counselling, patient education, prophylactic antacids, calcium supplements, and screening for functional independency. Six (60%) trials reported improved process of care [28,29,33,41,46,52] including one trial demonstrating higher ordering rates for pneumococcal vaccination (35.8% of patients in the intervention group versus 0.8% of those in the control group, *p*<0.001), influenza vaccination (51.4% versus 1.0%, *p*<0.001), prophylactic heparin (32.2% versus 18.9%, *p*<0.001), and prophylactic aspirin at discharge (36.4% versus 27.6%, *p*<0.001) in a teaching USA hospital [29]. Conversely, Overhage *et al. *[42] showed that a CCDSS for 22 preventive care measures in hospital inpatients did not change clinicians' actions for such measures. Only two studies assessed patient outcomes, but neither showed effects [16,34].

### Screening and management of CV risk factors (9 trials)

CCDSSs helped clinicians detect and treat dyslipidaemia, diabetes, smoking, obesity, hypertension, and renal diseases as well as calculating coronary risk factor profiles. All nine trials reported improved process of care of which three targeted screening and treatment of dyslipidaemia in primary care [18,19,27,56]. Five trials reported patient outcomes; three showed positive effects [17,38,43-45], and two [27,54,55] showed no effects.

### Screening and management of mental health-related conditions (6 trials)

Studies in this category covered various CCDSSs for screening and management of mental health conditions in primary, secondary, and tertiary care settings. Only one trial [47] used cluster randomization (see Additional file 1 Table S1) and all but one trial [51] were conducted in a single site. In all six trials, the CCDSSs were stand-alone systems (see Additional file 2 Table S2), and four trials included patient-completed computer-based instruments [15,37,48] or paper-based post intervention surveys [47]. Two trials showed positive effects in process of care, including Ahmad et al. [15] who reported that a CCDSS increased opportunities to discuss intimate partner violence in primary care (adjusted relative risk [RR], 1.4; 95% CI, 1.1 to 1.9) and increased its detection (adjusted RR, 2.0; 95% CI, 0.9 to 4.1). Three trials reported on patient outcomes including one with positive [51] and two with no effects [37,47].

### Vaccination (3 trials)

CCDSSs for tetanus, hepatitis, pneumococcal, measles, and influenza vaccinations in children, adults, and the elderly in primary care only improved influenza vaccination among the elderly in one trial [25]. All trials compared 'usual care' with CCDSS alone [25,32] or in addition to an educational session [1], and no trials assessed patient outcomes.

### Other preventive care activities (3 trials)

Two trials reported improved process of care [36], including one that also assessed patient outcomes but found no effects [59]. All studies in this category compared CCDSSs with 'usual care' although in one study, all providers were educated on the importance of HIV screening and trained on CCDSS functions [50]. Lafata *et al. *[36] showed that, among insured women 65 to 89 years of age, reminders mailed to patients, either alone or with physician prompts, improved osteoporosis screening and treatment rates. Zanetti *et al. *[59] showed improved intraoperative redose of prophylactic antibiotic, but it was underpowered to demonstrate effects on patient outcomes.

### Costs and practical process related outcomes (see Additional file 5, Table S5)

Costs of developing, implementing, and maintaining a CCDSS were partly reported in 6/41 (15%) trials [16,27,33,46,54,55,57,58]. Among these six studies, when a CCDSS was used, two found costs of care were significantly less [27,46], three yielded increased cost of care [16,33,54,55], and one showed varied cost minimization data [57,58]. Rosser *et al. *[46] did not report detailed costs, although the physician reminder was reported to be the most cost-effective method of improving preventive services, followed by letter reminder, and telephone reminder. Cobos *et al. *[27] showed that a CCDSS for management of patients with dyslipidaemia including those without coronary heart disease had no effects on lipid profiles, but saved 24.9% in treatment cost per patient and 20.8% in total costs, including costs for physician visits, laboratory analyses, and lipid-lowering drugs. Apkon *et al. *[16] showed a difference of US $91 more patient resource usage (ambulatory visits, laboratory tests, diagnostic imaging, and pharmacy use) for multiple preventive care procedures in the CCDSS group than usual-care group. Frame et al. showed that a CCDSS for multiple preventive procedures did not increase revenue generation or the number of office visits to a fee-for-service clinic despite its positive effects on provider compliance to such activities [33], and Unrod *et al*. found implementation costs for CCDSS, including equipment, training, and staff costs, increased costs for smoking cessation counselling [54,55]. Wilson *et al*. presented software development costs and the marginal cost for each additional compact disc [57,58].

Only two (5%) trials reported CCDSS adverse events; one demonstrated greater risk for over treatment than for under treatment in dyslipidaemia because all patients were screened, including low-risk patients who would not normally be screened [18,19]. Zanetti *et al. *[59] reported four in 449 (1%) inappropriate alerts to redose prophylactic antibiotics during cardiac surgery and one unnecessary intraoperative redosing [59].

Six (15%) trials reported on provider satisfaction with CCDSSs [15,16,30,40,49,50] including two trials [30,49] on cancer screening where most providers were satisfied with CCDSSs use. Only Apkon *et al. *[16] reported provider and patient satisfaction when a CCDSS was used, but showed no significant differences between groups in patient satisfaction results and mixed providers' satisfaction within the group using a CCDSS for 12 preventive care interventions.

## Discussion

We added 17 new trials to our previous review [6], and synthesised the evidence from 41 RCTs of CCDSSs for PPC. Forty of 41 trials examined process of care for which the majority of CCDSSs, 25/40 (63%), were effective using a dichotomous measure of effect. Recent trials more often reported patient outcomes (14/41 (34%) versus 1/24 (4%) study in our 2005 review [6]), but these outcomes were mostly surrogates (*e.g*., cholesterol level) rather than major patient outcomes.

For CCDSSs showing positive effect, it is important to be cautious about ascribing positive effects solely to CCDSSs [79] because most interventions included multiple components, such as educational sessions for clinicians and outreach to patients, and all trials were unblinded. For CCDSSs showing no effect, control-group clinicians often received training on the condition and recommended care. These 'educated' participants may have diluted intervention effects. Moreover, the reality of clinical practice, such as patients' varied adherence to recommendations, deficient follow-through by healthcare services, and long waiting lists for preventive care procedures [34], may have reduced intervention effects. In short, interventions directed at provider behaviors are bound to have limited effect on actions that also require patient adherence and service support to realize such actions.

Our review found that CCDSSs for PPC rarely reported cost-effectiveness and harm assessments. Within the 6/41 (15%) RCTs reporting costs, the majority only performed cost comparisons of interventions, not cost-effectiveness analysis [80]. Reporting was often incomplete, focused mainly on the CCDSS operating expenses, and varied substantially in methods of calculating costs and items included in analyses. There also was limited reporting on CCDSS-caused harm. The paucity of cost-effectiveness and harm analyses in PPC-related CCDSS studies is consistent with the current literature [9,81].

Findings in this review may not be generalised to low- and middle-income countries because all included trials were conducted in high-income countries, the CCDSS costs and context-related data were incompletely reported, and many CCDSSs were integrated with electronic health records. These factors may hinder implementation or scaling up of CCDSSs in resource scarce settings, and it remains unclear if and how such settings might achieve similar benefits and at what costs. Moreover, patients' and organizational culture and values may influence implementation of CCDSSs' recommendations in different settings. That said, until CCDSSs show more reliable and substantial effects, delays in studies and implementation in resource-limited settings may be fortunate.

Our review endorsed the shift that trials of CCDSS have been making since 1976 [82] from single university-based practices, with medical residents as users, small numbers of patients, and covering a few interventions, to multiple settings, used by physicians and multi-professional teams, encompassing larger numbers of patients with multiple health conditions and interventions. It also supported that assessment of patient outcomes [83], associated costs, and safety have seen limited increases [6,84,85].

### Study strengths and limitations

We built on our previous review by including only RCTs published in any language, and using duplicate study identification, data abstraction, and study evaluation. Our current focus on RCTs provided a more scientifically robust estimate of CCDSS effectiveness, although the potential for publication bias was not assessed. We confirmed our abstractions with primary authors. We collaborated with clinical decision-makers in extracting and analyzing data, and formulating and disseminating findings. We considered the methodological rigor of trials. We could not use meta-analysis to pool effect sizes because included RCTs presented a considerable variety of systems and outcomes. The vote counting approach that we used to summarize study results does not take into account the size or quality of individual studies.

Our decision-maker partners indicated concerns regarding insufficient reporting on infrastructure and contextual factors in which CCDSSs were evaluated, including impact on clinician workflow and the interoperability across different systems. An assessment of available data across all studies in the review set (166 RCTs) is in progress.

Although trial methods improved over time, our review was hampered by the limitations of the primary studies. CCDSSs should target processes of care that have already been shown to be validly related to improved patient outcomes, but not all studies reported the validity of the targeted processes. In addition, most trials did not assess patient outcomes, and even the trials that did were too small to detect clinically important effects. Further, information on study duration was often missing, limiting our ability to assess sustainability of CCDSS effects.

## Conclusions

Our review found a growing number of RCTs that assessed a wide variety of CCDSSs designed to improve PPC. To date, the included trials showed good evidence for the effectiveness of CCDSSs for screening and treatment/management of dyslipidaemia in primary care, and mixed evidence for CCDSSs in screening of cancer and mental health-related conditions, multiple preventive care activities, vaccination, and other preventive care interventions. Although CCDSSs for PPC did not seem to cause any serious adverse effects and may reduce some costs of care, most trials did not assess or report these findings. Despite the cumulative knowledge of CCDSSs, it is still not possible to draw definite conclusions on their effectiveness, especially for patient outcomes, because of heterogeneity in systems, settings, and outcomes assessed.

## Competing interests

RBH, NLW, RJS, JAM, NMS, LWK, TN, JP received support through the Canadian Institutes of Health Research Synthesis Grant: Knowledge Translation KRS 91791for the submitted work; RJS is the owner of Fig.P Software Incorporated, which develops and sells a chronic disease management system that is not a subject of this review. RBH is acquainted with several CCDSS developers and researchers, including authors of papers included in this review.

## Authors' contributions

RBH was responsible for study conception and design; acquisition, analysis and interpretation of data; drafting and critical revision of the manuscript; obtaining funding; and study supervision. He is the guarantor. NMS acquired, analysed, and interpreted data; drafted and critically revised the manuscript; and provided statistical analysis. RJS analysed and interpreted data; and drafted and critically revised the manuscript. JAM acquired, analysed, and interpreted data; drafted the manuscript; and provided administrative, technical or material support. JP acquired data; drafted the manuscript; and provided administrative, technical, or material support. LWK and TN acquired data and drafted the manuscript. NLW acquired, analysed, and interpreted data; drafted the manuscript; provided administrative, technical, or material support; and provided study supervision. All authors have read and approved the final manuscript.

## Supplementary Material

Additional file 1 **Study methods scores for trials of primary preventive care**. Methods scores for the included studies. Click here for file

Additional file 2 **CCDSS characteristics for trials of primary preventive care**. CCDSS characteristics of the included studies. Click here for file

Additional file 3 **Study characteristics for trials of primary preventive care**. Study characteristics of the included studies. Click here for file

Additional file 4 **Results for CCDSS trials of primary preventive care**. Details results of the included studies. Click here for file

Additional file 5 **Costs and CCDSS process-related outcomes for trials of primary preventive care**. Cost and CCDSS process-related outcomes for the included studies. Click here for file
