# Supplementary material for: Computerized clinical decision support systems for primary preventive care: A decision-maker-researcher partnership systematic review of effects on process of care and patient outcomes
Source: Implement Sci. 2011 Aug 3;6:87. doi: 10.1186/1748-5908-6-87 (PMC3173370; doi:10.1186/1748-5908-6-87)
Supplement: Additional file 1 — Study methods scores for trials of primary preventive care. Methods scores for the included studies. [file 1748-5908-6-87-S1.DOCX]

**Additional file 1, Table S1. Study methods scores for trials of primary preventive care^a^**

| **Study** | **Allocation concealed ^b^** | **Cluster randomization** | **Adjustment for baseline differences** | **Objective outcome** | **Adequate follow-up** | **Total score** |
| --- | --- | --- | --- | --- | --- | --- |
| Barnett, 1983[17] | 0 | 0 | 1 | 2 | 1 | 4 |
| Rogers, 1984[43-45] | 0 | 0 | 2 | 2 | 0 | 4 |
| Tierney, 1986[52] | 0 | 2 | 0 | 2 | 2 | 6 |
| McPhee, 1989[39] | 0 | 1 | 2 | 2 | 2 | 7 |
| Chambers, 1991[25] | 2 | 1 | 1 | 2 | 0 | 6 |
| McPhee, 1991[40] | 0 | 1 | 2 | 2 | 2 | 7 |
| Ornstein, 1991[41] | 0 | 2 | 1 | 2 | 2 | 7 |
| Rosser, 1991[46] | 0 | 0 | 2 | 2 | 2 | 6 |
| Burack, 1994[20] | 2 | 0 | 2 | 2 | 2 | 8 |
| Frame, 1994[33] | 2 | 0 | 2 | 2 | 0 | 6 |
| Turner, 1994[53] | 0 | 2 | 0 | 2 | 1 | 5 |
| Rubenstein, 1995[47] | 0 | 2 | 1 | 2 | 2 | 7 |
| Burack, 1996[21] | 2 | 0 | 2 | 2 | 2 | 8 |
| Lewis, 1996[37] | 0 | 0 | 2 | 2 | 2 | 6 |
| Overhage, 1996[42] | 2 | 2 | 2 | 2 | 2 | 10 |
| Burack, 1997[22] | 2 | 0 | 2 | 2 | 2 | 8 |
| Burack, 1998[23] | 0 | 0 | 2 | 2 | 2 | 6 |
| Lowensteyn, 1998[38] | 0 | 2 | 2 | 2 | 0 | 6 |
| Flanagan, 1999[32] | 0 | 1 | 0 | 2 | 0 | 3 |
| Cannon, 2000[26] | 0 | 0 | 2 | 2 | 0 | 4 |
| Demakis, 2000[28] | 0 | 2 | 1 | 2 | 2 | 7 |
| Dexter, 2001[29] | 2 | 2 | 2 | 2 | 2 | 10 |
| Schriger, 2001[48] | 2 | 0 | 2 | 2 | 2 | 8 |
| Burack, 2003[24] | 2 | 0 | 2 | 2 | 2 | 8 |
| Filippi, 2003[31] | 0 | 1 | 2 | 2 | 2 | 7 |
| Zanetti, 2003[59] | 2 | 0 | 2 | 2 | 2 | 8 |
| Thomas, 2004[51] | 2 | 0 | 2 | 2 | 1 | 7 |
| Apkon, 2005[16] | 0 | 0 | 2 | 2 | 1 | 5 |
| Cobos, 2005[27] | 2 | 2 | 2 | 2 | 2 | 10 |
| Kenealy, 2005[35] | 2 | 2 | 2 | 2 | 2 | 10 |
| Wilson, 2005[57, 58] | 0 | 2 | 2 | 2 | 0 | 6 |
| Emery, 2007[30] | 2 | 2 | 2 | 2 | 2 | 10 |
| Lafata, 2007[36] | 2 | 2 | 2 | 2 | 1 | 9 |
| Unrod, 2007[54, 55] | 2 | 1 | 2 | 2 | 1 | 8 |
| Harari, 2008[34] | 2 | 0 | 2 | 2 | 1 | 7 |
| Van Wyk, 2008[56] | 2 | 2 | 2 | 2 | 2 | 10 |
| Ahmad, 2009[15] | 2 | 0 | 2 | 2 | 2 | 8 |
| Bertoni, 2009[18, 19] | 2 | 2 | 2 | 2 | 1 | 9 |
| Fiks, 2009[1] | 0 | 2 | 2 | 2 | 2 | 8 |
| Sequist, 2009[49] | 2 | 1 | 2 | 2 | 2 | 9 |
| Sundaram, 2009[50] | 0 | 1 | 2 | 2 | 2 | 7 |

^a^ Based on five individual items (score 2 = yes, 1 = partly, and 0 = no) and a summed total score (range 0 to 10). Because this review update included only randomized, controlled trials, the total score differs from that reported in the previous version of this review [6]: the item evaluating study type (randomized, quasi-randomized, or concurrent controls) has been replaced by one that evaluates use of concealed allocation (concealed, unclear, not concealed).

^b^ If allocation concealment is not readily apparent from the description provided in the published article, the primary author of the trial confirmed or indicated that allocation was concealed.
