# Supplementary material for: Computerized clinical decision support systems for primary preventive care: A decision-maker-researcher partnership systematic review of effects on process of care and patient outcomes
Source: Implement Sci. 2011 Aug 3;6:87. doi: 10.1186/1748-5908-6-87 (PMC3173370; doi:10.1186/1748-5908-6-87)
Supplement: Additional file 2 — CCDSS characteristics for trials of primary preventive care. CCDSS characteristics of the included studies. [file 1748-5908-6-87-S2.DOCX]

**Additional file 2, Table S2. CCDSS characteristics for trials of primary preventive care^a^**

| **Study** | **Design** | | | **Interface description** | | | | | **Data entry source** | | | | | | **Methods for delivery of recommendations** | | | | | | | **CCDSS users** | | | | | | **Other characteristics** | | | | |
| --- | --- | --- | --- | --- | --- | --- | --- | --- | --- | --- | --- | --- | --- | --- | --- | --- | --- | --- | --- | --- | --- | --- | --- | --- | --- | --- | --- | --- | --- | --- | --- | --- |
|  | **Stand Alone** | **Integrated with EMR** | **Integrated with CPOE** | Graphic user interface | User must type | Drop down menus | Drag and drop | Other interface | **Automated through EMR** | **Project staff** | **Existing staff** | **Practitioner/decision-maker** | **Patient** | **Other data entry** | Desktop/Laptop computer | E-Mail | PDA | Pager | Project staff | Existing non-prescribing staff | Other Methods | **Trainees** | **Physicians** | **Advanced Practice Nurses** | **Physician Assistants** | **Pharmacists** | **Other health professionals** | Pilot tested | Users trained | Feedback at time of care | CCDSS suggested diagnoses/ treatments/procedures | Authors as developers |
| Barnett, 1983[17] | **-** | **+** | **?** | ? | ? | ? | ? | ? | **+** | **-** | **+** | **-** | **-** | **-** | - | - | - | - | - | + | - | **-** | **+** | **+** | **-** | **-** | **-** | - | - | - | + | + |
| Rogers, 1984[43-45] | **-** | **+** | **-** | ? | ? | ? | ? | ? | **?** | **?** | **?** | **?** | **?** | **?** | - | - | - | - | - | + | - | **-** | **+** | **-** | **-** | **-** | **-** | - | - | + | + | + |
| Tierney, 1986[52] | **-** | **+** | **-** | ? | ? | ? | ? | ? | **-** | **+** | **-** | **-** | **-** | **-** | - | - | - | - | - | + | - | **+** | **+** | **-** | **-** | **-** | **-** | + | + | + | + | + |
| McPhee, 1989[39] | **-** | **+** | **-** | ? | ? | ? | ? | ? | **-** | **+** | **-** | **-** | **-** | **-** | - | - | - | - | + | - | - | **+** | **+** | **-** | **-** | **-** | **-** | + | + | + | + | + |
| Chambers, 1991[25] | **-** | **+** | **-** | - | - | - | - | ? | **-** | **-** | **+** | **-** | **-** | **-** | - | - | - | - | - | - | + | **+** | **+** | **-** | **-** | **-** | **-** | ? | ? | + | + | + |
| McPhee, 1991[40] | **+** | **-** | **-** | ? | ? | ? | ? | ? | **-** | **+** | **+** | **-** | **-** | **-** | - | - | - | - | - | + | - | **-** | **+** | **-** | **-** | **-** | **-** | + | + | + | + | + |
| Ornstein, 1991[41] | **-** | **+** | **-** | ? | ? | ? | ? | ? | **+** | **-** | **-** | **-** | **-** | **-** | - | - | - | - | - | + | - | **+** | **+** | **-** | **-** | **-** | **-** | - | - | + | + | - |
| Rosser, 1991[46] | **-** | **+** | **-** | ? | ? | ? | ? | ? | **+** | **-** | **-** | **-** | **-** | **-** | - | - | - | - | - | + | + | **+** | **+** | **-** | **-** | **-** | **-** | - | - | + | + | - |
| Burack, 1994[20] | **+** | **-** | **-** | ? | ? | ? | ? | ? | **-** | **+** | **-** | **+** | **-** | **-** | - | - | - | - | + | - | - | **-** | **+** | **-** | **-** | **-** | **-** | - | - | + | + | + |
| Frame, 1994[33] | **+** | **-** | **-** | ? | ? | ? | ? | ? | **-** | **-** | **+** | **+** | **-** | **-** | - | - | - | - | - | + | - | **-** | **+** | **-** | **+** | **-** | **-** | + | + | + | + | + |
| Turner, 1994[53] | **+** | **-** | **-** | ? | ? | ? | ? | ? | **-** | **-** | **+** | **-** | **-** | **-** | - | - | - | - | - | + | - | **-** | **+** | **-** | **-** | **-** | **-** | - | + | + | + | + |
| Rubenstein, 1995[47] | **+** | **-** | **-** | ? | ? | ? | ? | ? | **-** | **+** | **-** | **-** | **-** | **-** | - | - | - | - | + | - | - | **+** | **+** | **-** | **-** | **-** | **-** | + | + | + | + | + |
| Burack, 1996[21] | **+** | **-** | **-** | ? | ? | ? | ? | ? | **-** | **+** | **-** | **-** | **-** | **-** | - | - | - | - | + | - | - | **-** | **+** | **-** | **-** | **-** | **-** | - | - | + | + | + |
| Lewis, 1996[37] | **+** | **-** | **-** | ? | ? | ? | ? | ? | **-** | **-** | **-** | **-** | **+** | **-** | - | - | - | - | - | - | + | **-** | **+** | **-** | **-** | **-** | **-** | - | - | - | ? | + |
| Overhage, 1996[42] | **-** | **+** | **+** | ? | ? | ? | ? | ? | **+** | **-** | **-** | **-** | **-** | **-** | + | - | - | - | - | - | + | **+** | **+** | **-** | **-** | **-** | **-** | - | - | + | + | + |
| Burack, 1997[22] | **+** | **-** | **-** | ? | ? | ? | ? | ? | **-** | **+** | **-** | **+** | **-** | **-** | - | - | - | - | + | - | - | **-** | **+** | **-** | **-** | **-** | **-** | - | - | + | + | + |
| Burack, 1998[23] | **+** | **-** | **-** | ? | ? | ? | ? | ? | **-** | **+** | **-** | **-** | **-** | **-** | - | - | - | - | - | - | + | **-** | **+** | **-** | **-** | **-** | **-** | ? | ? | + | + | - |
| Lowensteyn, 1998[38] | **+** | **-** | **-** | - | ? | ? | ? | ? | **-** | **-** | **+** | **-** | **-** | **-** | - | - | - | - | + | - | - | **-** | **+** | **-** | **-** | **-** | **-** | - | - | - | ? | + |
| Flanagan, 1999[32] | **-** | **+** | **+** | + | - | - | - | - | **-** | **-** | **+** | **-** | **-** | **-** | + | - | - | - | - | - | - | **-** | **+** | **-** | **-** | **-** | **-** | - | + | + | + | + |
| Cannon, 2000[26] | **+** | **-** | **-** | + | + | ? | ? | + | **-** | **-** | **-** | **+** | **-** | **-** | + | - | - | - | - | - | - | **-** | **-** | **-** | **-** | **-** | **+** | ? | ? | - | + | + |
| Demakis, 2000[28] | **+** | **-** | **-** | - | + | - | - | + | **-** | **-** | **-** | **+** | **-** | **+** | + | - | - | - | - | + | - | **+** | **+** | **-** | **-** | **-** | **-** | - | + | + | + | + |
| Dexter, 2001[26] | **-** | **+** | **-** | ? | ? | ? | ? | ? | **+** | **+** | **+** | **-** | **-** | **-** | + | - | - | - | - | - | - | **+** | **+** | **-** | **-** | **-** | **-** | + | - | + | + | + |
| Schriger, 2001[48] | **+** | **-** | **-** | + | ? | ? | ? | + | **-** | **-** | **-** | **-** | **-** | **+** | - | - | - | - | - | - | + | **+** | **+** | **-** | **-** | **-** | **-** | + | + | + | + | - |
| Burack, 2003[24] | **+** | **-** | **-** | ? | ? | ? | ? | ? | **-** | **+** | **-** | **-** | **-** | **-** | - | - | - | - | + | - | - | **-** | **+** | **-** | **-** | **-** | **-** | - | - | + | + | + |
| Filippi, 2003[31] | **-** | **+** | **?** | ? | ? | ? | ? | ? | **+** | **-** | **-** | **-** | **-** | **-** | + | - | - | - | - | - | - | **-** | **+** | **-** | **-** | **-** | **-** | ? | + | + | + | ? |
| Zanetti, 2003[59] | **-** | **+** | **?** | ? | ? | ? | ? | + | **+** | **-** | **+** | **-** | **-** | **-** | + | - | - | - | - | + | - | **-** | **+** | **+** | **-** | **-** | **-** | ? | ? | + | + | + |
| Thomas, 2004[51] | **+** | **-** | **-** | ? | ? | ? | ? | ? | **?** | **?** | **?** | **?** | **?** | **?** | - | - | - | - | - | - | + | **-** | **+** | **-** | **-** | **-** | **-** | ? | ? | + | + | ? |
| Apkon, 2005[16] | **+** | **-** | **-** | ? | ? | ? | ? | ? | **-** | **-** | **-** | **+** | **+** | **-** | ? | ? | ? | ? | ? | ? | ? | **-** | **+** | **-** | **-** | **-** | **-** | + | + | - | + | - |
| Cobos, 2005[27] | **-** | **+** | **-** | ? | ? | ? | ? | ? | **+** | **-** | **-** | **-** | **-** | **-** | + | - | - | - | - | - | - | **-** | **+** | **-** | **-** | **-** | **-** | ? | ? | + | + | ? |
| Kenealy, 2005[35] | **-** | **+** | **?** | + | - | ? | ? | ? | **+** | **-** | **-** | **-** | **-** | **-** | + | - | - | - | - | - | - | **-** | **+** | **-** | **-** | **-** | **-** | - | + | + | + | - |
| Wilson, 2005[57, 58] | **+** | **-** | **-** | - | + | ? | ? | ? | **-** | **-** | **-** | **+** | **-** | **-** | + | - | - | - | - | - | - | **-** | **+** | **-** | **-** | **-** | **-** | + | + | + | + | ? |
| Emery, 2007[30] | **+** | **-** | **-** | ? | ? | ? | ? | ? | **-** | **-** | **-** | **+** | **+** | **-** | + | - | - | - | - | - | - | **-** | **+** | **+** | **-** | **-** | **-** | + | + | + | + | ? |
| Lafata, 2007[36] | **-** | **+** | **-** | ? | ? | ? | ? | ? | **+** | **-** | **-** | **-** | **-** | **-** | + | - | - | - | - | - | - | **-** | **+** | **-** | **-** | **-** | **-** | + | + | + | + | + |
| Unrod, 2007[54, 55] | **+** | **-** | **-** | ? | ? | ? | ? | ? | **-** | **-** | **-** | **-** | **+** | **-** | - | - | - | - | - | - | + | **-** | **+** | **-** | **-** | **-** | **-** | - | + | + | + | ? |
| Harari, 2008[34] | **-** | **+** | **?** | + | - | ? | ? | - | **+** | **-** | **+** | **-** | **-** | **-** | + | - | - | - | - | - | - | **-** | **+** | **-** | **-** | **-** | **-** | ? | + | + | + | + |
| Van Wyk, 2008[56] | **-** | **+** | **+** | + | + | ? | ? | + | **+** | **-** | **-** | **+** | **-** | **-** | + | - | - | - | - | - | - | **-** | **+** | **-** | **-** | **-** | **-** | + | + | + | + | + |
| Ahmad, 2009[15] | **+** | **-** | **-** | ? | ? | ? | ? | + | **-** | **-** | **-** | **-** | **+** | **-** | - | - | - | - | + | - | - | **-** | **+** | **-** | **-** | **-** | **-** | + | + | + | + | + |
| Bertoni, 2009[18, 19] | **+** | **-** | **-** | + | + | + | - | ? | **-** | **-** | **-** | **+** | **-** | **-** | - | - | + | - | - | - | - | **-** | **+** | **+** | **+** | **-** | **-** | - | + | + | + | + |
| Fiks, 2009[1] | **-** | **+** | **+** | ? | ? | ? | ? | ? | **+** | **-** | **-** | **-** | **-** | **-** | + | - | - | - | - | - | - | **+** | **+** | **+** | **-** | **-** | **-** | + | + | + | + | + |
| Sequist, 2009[49] | **-** | **+** | **+** | + | - | ? | - | + | **+** | **-** | **-** | **-** | **-** | **-** | + | - | - | - | - | - | - | **-** | **+** | **-** | **-** | **-** | **-** | + | + | + | + | - |
| Sundaram, 2009[50] | **-** | **+** | **-** | + | ? | + | - | + | **+** | **-** | **-** | **-** | **-** | **-** | + | - | - | - | - | - | + | **-** | **+** | **+** | **-** | **-** | **-** | - | + | + | + | + |

Abbreviations: CCDSS, computerized clinical decision support system; CPOE, computerized physician order entry system; EMR, electronic medical record; PDA, personal digital assistant.

^a^ Symbol key: +, characteristic present; -, characteristic absent; ~, characteristic sometimes present; ?, unstated or uncertain.
