# Supplementary material for: Computerized clinical decision support systems for primary preventive care: A decision-maker-researcher partnership systematic review of effects on process of care and patient outcomes
Source: Implement Sci. 2011 Aug 3;6:87. doi: 10.1186/1748-5908-6-87 (PMC3173370; doi:10.1186/1748-5908-6-87)
Supplement: Additional file 3 — Study characteristics for trials of primary preventive care. Study characteristics of the included studies. [file 1748-5908-6-87-S3.DOCX]

**Additional file 3, Table S3. Study characteristics for trials of primary preventive care ^a^**

| **Study (country)** | **Methods score ^b^** | **Funding source** | **Indication** | **No. of practitioners / patients** | **Setting ^c^ (No. of clinics / sites)** | **CCDSS intervention** | **Comparison** |
| --- | --- | --- | --- | --- | --- | --- | --- |
| **Cancer screening** | | | | | | | |
| Sequist 2009[49] (USA) | 9 | Public | Screening for colorectal cancer in primary care. | 110 / 21,860 | •Primary care (11/11) | EMR-embedded reminders to physicians and patients for colorectal cancer screening. Physician Intervention. Physicians received EMR-embedded colorectal cancer screening reminders during patient visits. Physicians could electronically order screening examinations through the reminder.  Patient Intervention. Patients received a mailing which included a letter, an educational pamphlet, a fecal occult blood test kit and phone number to call and schedule a flexible sigmoidoscopy or colonoscopy.  Randomization strategy. Physicians were randomized to receive the Physician Intervention or not. Each physician’s patients were then randomized to receive the Patient Intervention or not. | Usual care |
| Emery 2007[30] (UK) | 10 | Public | Management of familial cancer risk in primary care. | ... / 219 | •Primary care  (45/...) | All clinicians attended an education session on cancer genetics. The Genetic Risk Assessment on the Internet with Decision Support (GRAIDS) software was accessed by primary care clinicians for assessment and management of familial cancer risk. It provided pedigree-drawing tools and patient-specific management advice regarding a family history of breast/ovarian and colorectal cancer, and provided additional numerical risk information about breast cancer. | Current best practice. All general practitioners and practice nurses attended a 45-minute educational session on cancer genetics and received by mail a paper copy of the regional guidelines for familial breast/ovarian cancer and familial colorectal cancer. |
| Wilson 2005[57, 58] (Scotland) (UK) | 6 | Public | Computer support system for breast cancer genetic risk in a primary care setting. | 243 / 242 | •Primary care •Community-based clinic  (86/...) | CCDSS CD-ROM provided a referral guide based on the Scottish referral guidelines for breast, ovarian, and colorectal cancer. It included background information on these cancers, locally relevant information sheets, downloadable data from the referral guide, web links for practitioners and patients, and an e-mail link to contact the Cancer Genetics Service for advice. | Paper-based genetic cancer referral guidelines |
| Burack 2003[24] (USA) | 8 | Public | Mammography and pap smear tests for HMO primary care patients. | 20 / 2,471 | •Academic centre (3/...) | CCDSS generated physician and patient reminders for mammography and pap smear tests based on HMO administrative data for women ≥40 years of age. For the 20 participating physicians (two primary care, nine general internal medicine, and nine gynecology), the brightly-coloured physician reminder was placed in patient charts within two months of procedure due dates. The personalized patient reminder letter was mailed. Procedure due dates were one year after last procedure unless recommended otherwise (*e.g.,* two year period for mammography in women 40 to 49 years). Note: One of the two sites participated in authors’ 1994 trial. | Mammogram only reminders (process as for the combined intervention group) |
| Burack 1998[23] (USA) | 6 | Public | Pap smear screening in primary care. | 20 / 5,801 | •Primary care (3/3) | CCDSS generated pap smear reminders, triggered by patients' pap smear due dates, in accordance with HMO policy. Physician reminders were placed within the medical records by the research team two months before pap smear due date and removed after the test had been performed. Patients were mailed a personalized letter containing the rationale concerning pap smear as well as a brochure from the National Cancer Institute with information about pelvic examination and the pap smear procedure. | Usual care |
| Burack 1997[22] (USA) | 8 | Public | Mammography reminders for women in primary care. | 25 / 2,826 | •Primary care •Community-based clinic (3/...) | Full intervention included all components of the limited intervention plus computer-generated mammography appointment reminders. The system produced reminder forms, which were printed for physicians one month before mammography appointments and placed in patient charts by the research team. Note: This is a follow-up study to the 1994 publication and includes some patients from the 1994 study. | Limited intervention included physician and staff orientation and elimination of patient out-of-pocket expenses for mammography |
| Burack 1996[21] (USA) | 8 | Public | Screening mammography for women in two primary care sites. | 20 / 2,368 | •Subspecialty clinic •Primary care (2/2) | CCDSS operated by research team and provided one of three randomized mammography reminder options, generated off-site and based on HMO administrative data and mammography history in women ≥39.5 years of age: a) brightly-coloured, single-page physician reminders, which were placed in charts of women within one month of mammography due date during 1st year of study for 20 participating physicians (two primary care, nine general internal medicine, and nine gynecology); b) personalized patient reminder letters suggesting a physician visit mailed in 1st four months of study to patients due for mammography; or c) both physician and patient reminders. Mammography due date (unless recommended otherwise): one year after last mammogram in women > 49 years; two years after last mammogram in women 40 to 49 years; 1st day of study if no prior mammogram.  Note: One of the two sites participated in authors’ 1994 trial. | Usual care (no reminders) |
| Burack 1994[20] (USA) | 8 | Public | Mammography for women in primary care in inner cities. | 25 / 2,725 | •Primary care (5/...) | Full intervention included all components of the limited intervention plus a computerized mammography appointment reminder system operated by research staff. The system produced reminder forms, which were printed for physicians one month before mammography appointments and placed in patient charts, postcard reminders for patients one week before scheduled mammography appointments, and an appointment rescheduling system for patients unable to keep their appointments. | Limited intervention included a breast cancer awareness program for physicians and staff, a project-dedicated telephone line for mammography appointments and patient reminders of scheduled appointments, and reduction or elimination of patient out-of-pocket expenses for mammography |
| McPhee 1991[40] (USA) | 7 | Public | Cancer screening (digital rectal examination, stool occult blood, sigmoidoscopy, pelvic examination, Papanicolaou test, breast examination, mammography) and preventive counselling (smoking assessment and counselling, dietary assessment and counselling). | 40 / ... | •Primary care •Solo practice (.../...) | Research staff audited files and entered pre-intervention data into the Cancer Prevention Reminder System (CPRS). Subsequent patient data were entered by office staff. The CPRS generated physician and patient reports indicating current patient status and cancer prevention activities due, and office staff printed and attached the reminders to patient charts prior to visits. Patient education material was also available. | No intervention |
| McPhee 1989[39] (USA) | 7 | Public | Outpatient screening (stool occult blood, digital rectal examination, sigmoidoscopy, pelvic examination, Papanicolaou test, breast examination, mammography). | 62 / 1,936 | •Academic centre (1/1) | 3 x 2 study. 1 & 2. CCDSS generated reminders for cancer screening, based on audit and visit data entered by research staff. Research staff printed reminders and placed in patient charts prior to visits. Also randomized to provide education (mailed letter and pamphlets) to female patients on professional breast exams and mammography or not. 3 & 4. Manual audit and feedback with or without patient education. 5. Patient education alone. | No reminders and no patient education |
| **Multiple preventive care activities** | | | | | | | |
| Harari  2008[34]  (England) (UK) | 7 | Public | Primary preventative care and screening for functionally independent, community-dwelling, geriatric patients in primary care. | 26 / 2,503 | •Primary care •Community-based clinic (4/4) | Self-administered health risk appraisal questionnaire leading to computer-generated individualized feedback to participants and general practitioners as part of primary care practice information technology systems. Patient feedback was a 20 to 35 page personalized report which included advice on modifying health risks, a prevention checklist, sources of support, and information on when to seek medical or other advice. Feedback to general practitioners included a one page clinical information summary. | Usual care. All general practitioners and practice nurses received an educational session on preventative care. |
| Apkon 2005[16] (USA) | 5 | Public | Screening, preventive care, and management of acute or chronic conditions for patients receiving routine ambulatory care in military facilities. | 12 / 1,902 | •Hospital outpatients •Community-based clinic (3/3) | CCDSS (Problem-Knowledge Couplers) were incorporated into an EMR system and used patient and provider responses to structured questions (generally complaint-specific) and a medical knowledge database to provide suggestions for patient care, including diagnosis and treatment. Suggestions were based on national organization recommendations (e.g., Agency for Healthcare Research and Quality). Patients entered data into the system with assistance from a coordinator not associated with the study. | Usual care |
| Dexter 2001[29] (USA) | 10 | Public | Preventive therapies in hospital inpatients. | 202 / 3,416 | •Academic centre •Hospital inpatients (.../...) | CCDSS provided guideline-based reminders for preventative care procedures (pneumococcal vaccination, influenza vaccination, prophylactic entericoated aspirin for CV disease, and prophylactic subcutaneous heparin for thromboembolic events) to physicians and medical students. | Usual care |
| Demakis  2000[28] (USA) | 7 | Public | Screening, monitoring, and counselling in accordance with predefined standards in ambulatory care. | 275 / 12,989 | •Other •Academic centre •Hospital outpatients (12/12) | Residents received CCDSS-generated reminders relating to 13 prespecified standards of care in two ways. 1) On entering a patient name into a computer terminal in the examining room, applicable reminders were automatically displayed in bold letters. 2) Applicable reminders were printed on the standard patient health summary which is attached to patient charts at visits. | Control residents only received the standard health summaries without the reminders. |
| Overhage 1996[42] (USA) | 10 | Public | Compliance with 22 US Preventive Services Task Force preventive care measures for hospital inpatients, including cancer screening, preventive screening and medications, diabetes care reminders, and vaccinations. | 78 / 1,622 | •Academic centre •Hospital inpatients (1/1) | CCDSS was incorporated into the EMR and order-entry system and used data from these sources to generate reminders for 22 preventive care measures. CCDSS ran overnight and provided reminders to physicians in two ways: printed at the top of daily patient reports, and displayed at the bottom of the workstation screen in red when physicians entered orders for patients. Physicians could accept or reject orders generated by the reminder program. | Usual care |
| Frame 1994[33] (USA) | 6 | Public | Cancer screening, CV disease preventive screening, identification of at-risk behaviour, patient education, and vaccination in a rural primary care setting. | 12 / 1,324 | •Primary care (5/...) | CCDSS generated physician reminders for 11 health maintenance procedures (including stool occult blood, Papanicolaou, breast examination, and mammogram tests; blood pressure, cholesterol, and body weight screening; and vaccination), based on health maintenance protocols and patient visit data recorded by physicians and entered by data entry staff. Reminders were placed at the front of patient charts annually, and patients also received telephone reminders. | Health maintenance procedures tracked using manual, paper flowcharts completed by physicians and kept at the front of patients’ charts. Physicians could request a telephone reminder for patients. |
| Turner 1994[53] (USA) | 5 | Public | Cancer screening (stool occult blood, Papanicolaou test, breast examination, mammogram) and influenza vaccination in primary care. | 44 / 740 | •Primary care •Solo practice (44/...) | Physicians received a computer with a 20-megabyte hard disk, and a CCDSS written in spreadsheet data software which generated a prompt sheet for health care activities: influenza vaccination, stool occult blood tests, pap smears, physician-performed breast exams, and mammograms. The prompt sheet was placed in front of patients' charts. | In control group patients were given prompt cards and instructed to show them to physicians at each visit. |
| Ornstein 1991[41] (USA) | 7 | Public | Use of preventive care services for adults in university-based family medicine clinic. | 49 / 7,397 | •Academic centre (1/1) | CCDSS generated reminders for five preventive care services (cholesterol measurement, fecal occult blood testing, mammography, pap smears, and tetanus immunization), based on computerized patient medical records. Reminders were delivered to physicians at patient visits (placed in patient record) (A), mailed to patients (B), or both (C). All practitioners received educational and administration services including quarterly audits of the percentage of patients in each physician’s practice that were up to date with the five preventive services and a health maintenance flow sheet placed in all adult patients’ medical records. | Educational and administrative interventions only (D) |
| Rosser 1991[46] (Canada) | 6 | Public | Cancer screening (Papanicolaou test), blood pressure measurement, assessment of smoking status, and vaccination (influenza, tetanus toxoid) in outpatients. | ... / 5,883 | •Academic centre (1/1) | CCDSS generated paper reminders for physicians or letter reminders for patients or lists of patients to receive telephone reminders when the patient was due for any of five screening procedures. | No reminders |
| Tierney 1986[52] (USA) | 6 | Public | Cancer screening (stool occult blood, Papinicolaou test, mammogram), pneumococcal vaccination, tuberculosis skin test, use of antidepressants, metronidazole for trichomonas, CV medications (β-blockers, long-acting nitrates, aspirin), prophylactic antacids, and calcium supplements for outpatients. | 135 / 6,045 | •Academic centre •Subspecialty clinic (1/4) | Thirteen identified preventive care protocols were randomly divided into two groups (A and B). CCDSS (as part of the Regenstrief Medical Record System) identified eligible patients who had not received protocol care and generated monthly feedback reports for physicians indicating any actions that should be taken for each patient. Physicians received reports on either A or B protocols and had to respond with one of five options (including ‘not applicable’) to each item on the report.  Physicians were also randomized to receive CCDSS-generated reminders for Group A or B protocols at patient visits. The reminders were generated the night before visits and placed in the patient clinic charts. | 2 x 2 factorial trial with physicians in the protocol A feedback group acting as controls for those in protocol B and vice versa |
| **Screening and management of CV risk factors** | | | | | | | |
| Bertoni 2009[18, 19] (USA) | 9 | Public | Guideline-consistent screening and treatment of dyslipidemia in primary care. | ... / 3,821 | •Primary care (59/59) | CCDSS ran on personal digital assistants given to providers (physicians, physician assistants, and nurse practitioners) in the intervention group. CCDSS generated a one-page report summarizing patient data, LDL-C level goals, and treatment recommendations, based on National Cholesterol Education Program Third Adult Treatment Panel III guidelines. Providers also received print copies of guidelines, education, and academic detailing. | Comparison group were given automatic blood pressure measurement devices, print copies of guidelines, education, and academic detailing. |
| Van Wyk 2008[56] (The Netherlands) | 10 | Public | Screening and treatment of dyslipidemia in primary care. | 80 / 92,054 | •Primary care •Solo practice •Community-based clinic (38/38) | There were two versions of the CCDSS: on-demand and automatic alerting, both integrated with an EHR and based on guidelines from the Dutch College of General Practitioners. The CCDSS generated patient-specific recommendations for preventative care and displayed them on an interactive patient overview screen in the EHR. With the on-demand CCDSS, users had to actively initiate the overview screen. With the automatic alerting CCDSS, recommendations were automatically displayed to users. | Usual care |
| Unrod 2007[54, 55] (USA) | 8 | Public | Computerized intervention designed to increase smoking cessation counselling and quit rates within a primary care setting. | 70 / 465 | •Primary care (.../4) | CCDSS used to increase physician smoking cessation counselling using a patient-tailored expert-system report. Patients were classified by level of readiness to quit, nicotine dependence level, measurement on Pros and Cons smoking association scale, self-efficacy scale, patient smoking/cessation history, and by existing medical conditions. | Usual care |
| Cobos 2005[27] (Spain) | 10 | Private | Management of patients with hypercholesterolemia in primary care. | ... / 2,221 | •Primary care (42/44) | CCDSS generated recommendations for hypercholesterolemia therapy, follow-up visit frequency, and laboratory test ordering, based on patient data entered by physicians, including CV risk and LDL-C goals. Recommendations were adapted from the European Society of Cardiology and other societies for Hypercholesterolemia Management’s (ESCHM). Guidelines. Physicians could adopt or ignore the recommendations. The intervention included availability of patient education promotions such as tablecloths and refrigerator magnets. | Usual care |
| Kenealy 2005[35] (New Zealand) | 10 | Public | Screening for diabetes in outpatients attending a family practice. | 107 / 5,628 | •Primary care •Solo practice •Community-based clinic (66/...) | 2 x 2 trial. CCDSS showed a slowly flashing icon on the task bar when physicians opened eligible patient files. On clicking this icon, a brief message appeared suggesting screening for diabetes. The icon flashed each time the patient record was opened until the practitioner marked the task as “complete”.  Patients were also randomized to receive patient reminders or not. | Usual care (no reminders) |
| Filippi 2003[31] (Italy) | 7 | ... | Prescribing of anti-platelet medications to diabetic primary care patients with ≥1 additional CV risk factor. | 300 / 15,343 | •Primary care (.../...) | CCDSS was integrated into a standard clinical practice management system and displayed an electronic reminder when general practitioners opened medical records of diabetic patients ≥30 years of age. Physicians could deactivate the reminder. A letter summarizing practice guidelines, including the benefits of anti-platelet drugs in high-risk diabetics, was also sent to practitioners. | Usual care plus the letter summarizing practice guidelines |
| Lowensteyn 1998[38] (Canada) | 6 | Public, Private | Calculating coronary risk factor profile for outpatients. | 253 / 958 | •Primary care (24/...) | Computerized system used mailed physician and patient-reported data to produce an individualized coronary risk profile. The profile was mailed back to the physician and a copy given to the patient after physician interpretation. | Physicians used clinical judgment at initial visits to identify patients at high-risk for coronary events. Computerized coronary risk profiles could be provided after three months if patients were clinically reevaluated. |
| Rogers 1984[43-45] (USA) | 4 | Public | Management of hypertension, obesity, and renal disease in outpatients. | ... / 484 | •Academic centre •Subspecialty clinic (1/1) | CCDSS summarized patient demographics, status, and health records and made suggestions based on deficiencies in patient care. The eight page patient medical summary (Northwestern University Computerised Medical Record Summary System, [NUCRSS]) was available to the physician at each visit. | Usual care |
| Barnett 1983[17] (USA) | 4 | Public | Follow-up for patients with newly-identified elevated blood pressure in an acute care setting. | ... / 115 | •Academic centre •Primary care (1/1) | CCDSS embedded in HER (COSTAR, no definition of this is given in article) sent reminders and encounter forms on which the target date of next visit could be recorded for physicians when patient with initial hypertension reading (diastolic measurement 100 to 120) was not followed by two repeat visits that included blood pressure measurement. Reminders continued until an appropriate follow-up occurred. | Reminders were not provided to physicians |
| **Screening and management of mental health-related conditions** | | | | | | | |
| Ahmad 2009[15] (Canada) | 8 | Public | Screening for intimate partner violence in primary care. | 11 / 314 | •Academic centre •Hospital outpatients •Primary care (1/1) | CCDSS used to screen for intimate partner violence at a multiphysician hospital-affiliated, academic family practice clinic. The program administered a survey to patients and generated risk reports for physicians and recommendation sheets for patients. | Usual care with no screening before the consultation |
| Thomas 2004[51] (UK) | 7 | Public | Identification and management of outpatients with anxiety and depression. | ... / 762 | •Primary care (5/5) | Patient-specific computerized guidelines along with a computer generated report of psychiatric symptoms, probable psychiatric diagnosis, social impairment, major life events, likely suicide risk, and patient-specific treatment recommendations were delivered to physicians. | Usual care with locally agreed-upon guidelines |
| Schriger 2001[48] (USA) | 8 | Private | Psychiatric interview and diagnosis in the emergency department. | 104 / 259 | •Academic centre •Hospital inpatients •Emergency Department (1/1) | Eligible patients completed a self-administered computer interview (Primary Care Evaluation of Mental Disorders [PRIME-MD]) in the waiting room. PRIME-MD screened for seven domains: mood disorder, anxiety disorder, alcohol abuse, eating disorder, obsessive compulsive disorder, phobia, and somatization disorder. When screening was positive for a particular domain, the CCDSS presented additional questions to establish or reject diagnoses within that domain. A report that indicated presence or absence of each psychiatric diagnosis considered was attached to the front of the physician section of the medical record. | Eligible patients completed the PRIME-MD in the waiting room but results were not given to physicians. |
| Cannon 2000[26] (USA) | 4 | ... | Screening and diagnosis of mood disorder in an outpatient mental health clinic. | 4 / 78 | •Academic centre •Subspecialty clinic (1/1) | CCDSS (CaseWalker) produced daily lists for providers (clinical psychologist, registered nurse, social worker, or addiction therapist) of patients eligible for mood disorder screening. When the provider opted to process the guideline-based reminder, the system provided an interactive checklist used for diagnosing major depressive disorder according to DSM-IV criteria. The system scored the criteria and produced a progress note. | A paper checklist inserted into the assessment section of the paper medical record, containing the mood disorder screening test and the DSM-IV criteria. |
| Lewis 1996[37] (UK) | 6 | Public | Assessment of common mental disorders in primary care. | 8 / 681 | •Primary care (1/1) | Patients scoring >1 on the manually scored, self-report 12-item General Health Questionnaire (GHQ) completed a self-report computerized assessment for minor psychiatric disorders (PROQSY) using the revised Clinical Interview Schedule within seven days. Physicians reminded patients assigned to the PROQSY group to return within one week when the PROQSY assessment would be placed in patient charts. | Two groups of patients scoring >1 on the GHQ: a) GHQ was placed in patient chart before consult or b) no additional data placed in chart (usual care). |
| Rubenstein 1995[47] (USA) | 7 | Public | Computer-generated feedback designed to identify and suggest management for functional deficits in primary care. | 73 / 557 | •Academic centre •Primary care (1/1) | After physicians attended a ½ hour education session, they started to receive CCDSS-generated patient-specific functional status reports, which included bar graphs, summarized functional deficits and assessment findings, and provided problem-specific resource and management suggestions. The reports were attached to the front of each new patient’s medical record. Physicians received a booster education session after three months, and patients were mailed post-intervention functional status surveys six months after their enrolment. | Usual care |
| **Vaccinations** | | | | | | | |
| Fiks 2009[1] (USA) | 8 | Public | Influenza vaccination for children and adolescents with asthma in primary care. | ... / 11,919 | •Primary care (20/...) | EHR-based alerts were generated for influenza vaccination in children 5 to 19 years of age, based on recommendations of the Advisory Committee on Immunization Practices. Bolded and highlighted alerts appeared at the top of the computer screen when an EHR encounter form was opened for an eligible patient, along with a link for ordering vaccine. An influenza education session, with information on the alert system, was provided by two expert primary care paediatricians. | Routine care and an influenza education session (without information on the alert system) provided by two expert primary care paediatricians |
| Flanagan 1999[32] (USA) | 3 | ... | Tetanus, hepatitis, pneumococcal, measles, and influenza vaccination for adult primary care outpatients. | 233 / 817 | •Academic centre •Primary care (.../...) | Computer used patient age and vaccine history to recommend or flag for consideration various vaccines. Physician could override recommendation or order vaccine or other vaccines. | Usual care |
| Chambers 1991[25] (USA) | 6 | ... | Influenza vaccination in university-based primary care practice. | 30 / 686 | •Academic centre •Primary care (1/1) | CCDSS-generated reminders identified patients eligible for influenza vaccination based on physician-determined rules and patient contact history (recorded by physicians and entered in the patient database after each visit by office staff). Reminders were always or sometimes included in clinical encounter forms placed in patient charts before visits. | Usual care (no reminders) |
| **Other preventive care activities** | | | | | | | |
| Sundaram 2009[50] (USA) | 7 | Public | Risk assessment and screening for HIV in primary care. | 32 / 26,042 | •Primary care (5/5) | EMR-embedded CCDSS used patient data to generate reminders for HIV risk assessments and HIV testing. Physicians and registered nurse practitioners received electronic reminders to assess HIV risk or test for HIV when they were in the patient medical record system or paper reminders on laboratory result and medication print outs. The reminders included a link to the Centres for Disease Control and Prevention guideline for HIV testing and counselling. Electronic reminders appeared each time a patient’s medical record was opened until the practitioner completed an interactive dialog box. Providers also received electronic and paper feedback on their actions to resolve reminders every two months. All providers received an educational session on the importance of HIV screening and watched a demonstration of the CCDSS reminders. | Usual care. All providers received an educational session on the importance of HIV screening and were given a demonstration of the computer-based clinical reminders. |
| Lafata 2007[36] (USA) | 9 | Private | Osteoporosis screening for female outpatients aged 65 to 89 in a primary care setting. | 123 / 10,354 | •Primary care •Community-based clinic (15/15) | Patient-mailed reminders and physician prompts were used to improve osteoporosis screening. Patient-mailed reminders consisted of initial and follow-up information about osteoporosis, patient risk factors, and screening information. Women receiving screening were also mailed information regarding injury prevention and tips. Physician prompts included a computerized EMR prompt and three to six month post-screen mailing reminder. | Patient mailed reminders and usual care |
| Zanetti 2003[59] (USA) | 8 | Public | Redosing of prophylactic antibiotics during prolonged cardiac surgery. | ... / 447 | •Academic centre •Hospital inpatients (1/1) | CCDSS provided an automated audible alarm and visual intraoperative alert on the operating room computer console for physicians to redose prophylactic antibiotics during cardiac surgery at 225 minutes after administration of preoperative antibiotics. A reply was required to clear the display. If planned redosing was indicated, a new alarrn and alert were issued after 30 minutes and the circulating nurse was required to indicate whether a follow-up dose of antibiotics had been administered. | Usual care |

Abbreviations: CCDSS, computerized clinical decision support system; CD-ROM, compact disk – read only memory; CV, cardiovascular; DSM-IV, Diagnostic and Statistical Manual of Mental Disorders, 4^th^ edition; EHR, electronic health record; EMR, electronic medical record; HIV, human immunodeficiency virus; HMO, health maintenance organization; LDL-C, low-density lipoprotein cholesterol; PROQSY, Programmable Questionnaire system.

^a^Ellipses (…) indicate item was not assessed.

^b^Based on five individual items (score 2 = yes, 1 = partly, and 0 = no) and a summed total score (range 0 to 10). Because this review update included only randomized, controlled trials, the total score differs from that reported in the previous version of this review [6]: the item evaluating study type (randomized, quasi-randomized, or concurrent controls) has been replaced by one that evaluates use of concealed allocation (concealed, unclear, not concealed).

^c^ Diabetes clinic is an example of a subspecialty clinic.
