# Supplementary material for: Computerized clinical decision support systems for primary preventive care: A decision-maker-researcher partnership systematic review of effects on process of care and patient outcomes
Source: Implement Sci. 2011 Aug 3;6:87. doi: 10.1186/1748-5908-6-87 (PMC3173370; doi:10.1186/1748-5908-6-87)
Supplement: Additional file 4 — Results for CCDSS trials of primary preventive care. Details results of the included studies. [file 1748-5908-6-87-S4.DOCX]

**Additional file 4, Table S4. Results for CCDSS trials of primary preventive care**

| **Study**  **(country)** | **Process of care outcomes** | **CCDSS vs. control** | **Patient outcomes** | **CCDSS vs. control** | **CCDSS process of care effect^b^** | **CCDSS patient effect^b^** |
| --- | --- | --- | --- | --- | --- | --- |
| **Cancer screening** | | | | | | |
| Sequist 2009[49] (USA) | Patient mailed reminder vs. control (regardless of physician intervention) (N=10930 vs. N=10930) **1. N (%) of individual tests performed for patient mailed reminder vs. control, % difference, (95% CI), p-value**  **1a. FOBT (primary)** 1b. Positive FOBT result (among FOBT) 1c. Follow-up colonoscopy (among positive FOBT result)  **1d. Flexible sigmoidoscopy (primary) 1e. Colonoscopy (primary)** 1f. % order for colonoscopy placed during the study  **Physician reminder vs. control (regardless of patient reminder) (N=10912 vs. N=10948)  2. N (%) of individual tests performed, % difference, (95% CI), p-value  2a. FOBT (primary)** 2b. Positive FOBT result (among FOBT) 2c. Follow-up colonoscopy (among positive FOBT result)  **2d. Flexible sigmoidoscopy (primary) 2e. Colonoscopy (primary)** 2f. % order for colonoscopy placed during the study  Patient mailing (with vs. without patient mailing, regardless of physician intervention or not) (N=10930 vs. N=10930) (Subgroup analyses not prespecified.) 3. % of patients who completed screening by grouping, % difference, (95% CI), p-value  3a. All patients 3b. Patients aged 50-59 3c. Patients aged 60-69 3d. Patients aged 70-80  3e. Trend towards effectiveness in older patients 3f. Females 3g. Males 3h. 0 primary care visits  3i. 1-2 primary care visits 3j. ≥3 primary care visits  Physician Reminder (with vs. without physician reminder, regardless of patient intervention of not) (Subgroup analyses not prespecified.) 4. % of patients who completed screening by grouping, % difference, (95% CI), p-value 4a. All patients  4b. Patients aged 50-59 4c. Patients aged 60-69 4d. Patients aged 70-80  4e. Females 4f. Males 4g. 0 primary care visits  4h. 1-2 primary care visits 4i. ≥3 primary care visits  5a. Screening rates by physician reminder and patient mailing vs. patient mailing vs. physician reminder vs. neither reminder nor mailing. 5b. Interaction between patient intervention and physician intervention, % difference between combined intervention and sum of individual intervention, (95% CI), p-value | 1a. 2779 (25.4%) vs. 2225 (20.4%), 5.1% (3.8 to 6.3), *P* < .001 1b. 47/2779 (1.7%) vs. 12/2225 (0.5%), 1.2% (0.6 to 1.7), *P* <.001 1c. 33/47 (70.2%) vs. 10/12 (83.3%), -11.9% (-37.9 to 14.1), *P* = .36 1d. 11 (0.1%) vs. 9 (<0.1%), 0.0% (-0.1 to 0.1), *P* = .66 1e. 2014 (18.4%) vs. 1933 (17.7%), 0.7%  (-0.3 to 1.8), *P* = .17 1f. 31.8% vs. 30.9%, *P* = .12  2a. 2505 (23.0%) vs. 2499 (22.8%), 0.1% (-5.5 to 5.7), *P* = .96 2b. 27/2505 (1.1%) vs. 32/2499 (1.3%), -0.2% (-0.8 to 0.4), *P* = .52 2c. 21/27 (77.8%) vs. 22/32 (68.8%), 7.8% (-15.4 to 31.0), *P* = .50 2d. 10 (<0.1%) vs. 10 (<0.1%), 0.0% (-0.1 to 0.1), *P* = .99 2e. 2056 (18.8%) vs. 1891 (17.3%), 1.6%  (-0.7 to 3.9), *P* = .18 2f. 33.1% vs. 29.6%, *P* =.004  3a. 44% vs. 38.1%, 5.8% (4.5 to 7.1), *P* <.001 3b. 42.1% vs. 38.4%, 3.7% (2.0 to 5.5), *P* <.001 3c. 45.4% vs. 38.0%, 7.3% (4.5 to 10.1), *P* <.001 3d. 47.4% vs. 37.3%, 10.1% (7.0 to 13.2), *P* <.001 3e. *P* = .01 3f. 44.3% vs. 38.6%, 5.7% (4.0 to 7.4), *P* <.001  3g. 43.5% vs. 37.5%, 6.0% (4.1 to 7.9), *P* <.001 3h. 19.6% vs. 15.6%, 3.9% (2.2 to 5.6), *P* <.001 3i. 55.6% vs. 49.0%, 6.6% (4.7 to 8.4), *P* <.001 3j. 59.5% vs. 52.3%, 7.1% (4.4 to 9.8), *P* <.001  4a. 41.9% vs. 40.2%, 1.6% (-2.7 to 5.9), *P* = .47 4b. 40.9% vs. 39.7%, 1.0% (-3.2 to 5.1), *P* = .64 4c. 43.2% vs. 40.4%, 2.7% (-2.4 to 7.8), *P* = .29 4d. 43.4% vs. 41.5 %, 2.0% (-3.8 to 7.8), *P* = .50 4e. 42.8% vs. 40.2%, 2.2% (-2.6 to 7.1), *P* = .36 4f. 40.8% vs. 40.1%, 0.7% (-4.7 to 6.2), *P* = .79 4g. 19.1% vs. 16.0%, 3.0% (-1.1 to 7.2), *P* = .15 4h. 53.2% vs. 51.5%, 1.6% (-3.8 to 7.1), *P* = .56 4i. 59.5% vs. 52.7%, 6.0% (-0.5 to 12.5), *P* = .07  5a. 44.2% vs. 43.7% vs. 39.6% vs. 36.7% 5b. -0.6% (-1.2% to 0.1%), *P* = .08 | **Patient mailed reminder vs. control (regardless of physician intervention)**  **1. N (%) of pathologic findings, % difference, (95% CI), p value (secondary)  1a. Colonic adenoma 1b. Colorectal cancer**  **Physician reminder vs. control (regardless of patient reminder)**  **2. N (%) of pathologic findings, % difference, (95% CI), p value (secondary)  2a. Colonic adenoma 2b. Colorectal cancer** | 1a. 622 (5.7%) vs. 568 (5.2%), 0.5% (-0.1 to 1.1), *P* = .10 1b. 19 (0.2%) vs. 15 (0.2%), 0.0% (-0.1 to 0.1), *P* = .43  2a. 650 (6.0%) vs. 540 (4.9%), 1.0% (-0.1 to 2.2), *P* = .09 2b. 17 (0.2%) vs. 17 (0.2%), 0.0% (-0.1 to 0.1), *P* = .99 | 0 | 0 |
| Emery 2007[30] (UK) | At practice level: 1.Mean referral rate per 10,000 registered patients per practice per year (SD); difference (95% CI), p-value (primary-outcome related) **2.Proportion (n, %) of referrals made to regional genetics clinic that were consistent with referral guidelines at 12 months, OR (95% CI), p-value (primary outcome). 2a. Breast and bowel cancer.** 2b. Breast cancer. 2c. Bowel cancer  Proportions with increased risk (determined by Regional Genetics Clinic) (prespecified): 2d. Breast and bowel cancer. 2e. Breast cancer. 2f. Bowel cancer. | 1. 6.2 (3.1) vs. 3.2 (2.8); 3.0 (1.12 to 4.8), *P* = .002  2a. 174/183 (95%) vs. 67/85 (79%), 5.2 (1.7 to 15.8), *P* = .006 2b. 99/107 (93%) vs. 44/60 (73%), 4.5 (1.6 to 13.1) 2c. 75/76 (99%) vs. 23/25 (92%), 6.5 (0.5 to 83.7) 2d. 90/132 (68%) vs. 40/53 (75%), 0.7 (0.3 to 1.5), *P* = .35 2e. 60/78 (77%) vs. 23/33 (70%), 1.4 (0.6 to.3.5) 2f. 30/54 (56%) vs. 17/20 (85%), 0.2 (0.1 to 0.8) | All predefined and assessed at referral. **1. Mean cancer worry score (lower is better) (SD), difference (95% CI), p-value 2.Risk perception score (lower is better) (SD), difference (95% CI), p-value 3. Accuracy of patient risk perception compared with Regional Genetics Clinic assessment; n (%) 3a. Accurate assessment.** 3b. Under-estimation. 3c. Over-estimation  **4.Knowledge about familial cancer (SD), difference (95% CI), p-value a.Colorectal b.Breast** | 1. 5.74 (3.04) vs. 7.18 (3.43), -1.44 (-2.64 to -0.23), *P* = .02 2. 4.99 (1.14) vs. 5.04 (0.88), -0.09 (0.34 to -0.51) 3a. 59 (68%) vs. 22 (55%) 3b. 18 (21%) vs. 9 (23%) 3c. 10 (11%) vs. 9 (23%)  4a. 5.50 (2.46) vs. 4.86 (3.30), 0.64 (-1.01 to 2.29), NS 4b. 5.77 (2.90) vs. 5.66 (2.78), 0.11 (-1.05 to 1.27), NS | + | 0 |
| Wilson 2005[57, 58] (Scotland, UK) | **Primary**  **Confidence in management of patient with family history of breast cancer concerns as measured by (intervention N=151, n (%) vs. control N=92, n (%), p-value) 1. Taking appropriate family history,  2. Knowing which patients need to be referred 3. Reassuring low-risk patients 4. Being able to answer questions**  The pre-determined secondary physician outcomes were changes in 5. Proportion (%) of referred patients with elevated genetic risk for post-intervention period. Intervention n/N (%) vs. control n/N (%), RR (95% CI) as probability that patients referred by intervention practices were at elevated risk.  6. Completeness of family history information in referral letters in the post-intervention period for intervention n/N (%) vs. control n/N (%). | 1. 91 (60%) vs. 56 (61%), *P* = .93 2. 60 (40%) vs. 30 (33%), *P* = .27 3. 85 (57%) vs. 48 (52%), *P* = .46 4. 35 (23%) vs. 20 (22%), *P* =.77 5. 49/85 (58%) vs. 14/29 (48%), 1.18 (0.88 to 1.37) 6. 102/108 (94%) vs. 37/37 (100%) | **Secondary**  **Changes in: 1. Perception of risk in post-intervention period as assessed by self-reported questionnaire : n (%); post-intervention period N=62 vs. N=18. 1a. Low perceived risk 1b. Elevated perceived risk RR (95% CI), p-value adjusted for clustering of patients within practice. 1c. High perceived risk 1d. Moderate perceived** **risk 2. Understanding of ‘incorrect’ breast cancer risk factors, intervention N=74 vs. control N=22, as measured by answers to the following questions: 2a. Stress is a major cause of breast cancer.** i. Agree/strongly agree ii. Disagree/strongly disagree iii. Not sure **2b. Having one relative with breast cancer always increases your risk considerably.** i. Agree/strongly agree ii. Disagree/strongly disagree iii. Not sure **2c. A healthy diet can prevent breast cancer.** i. Agree/strongly agree ii. Disagree/strongly disagree iii. Not sure **2d. Oral contraceptives can significantly increase the risk of breast cancer.** i. Agree/strongly agree ii. Disagree/strongly disagree iii. Not sure **2e. Minor injury to the breast can cause breast cancer.** i. Agree/strongly agree ii. Disagree/strongly disagree iii. Not sure | 1a. 12 (19.4%) vs. 4 (22.2%)  1b. 50 (80.6%) vs. 14 (77.8%), 1.04 (0.79 to 1.37), *P* = .79 1c. 11 (17.7%) vs. 2 (11.1%) 1d. 39 (62.9%) vs. 12 (66.7%)  2a. *P* = .57 2a.i. 23% vs. 22.7% 2a.ii. 29.7% vs. 40.9% 2a.iii. 47.3% vs. 36.4%  2b. *P* = .74 2b.i. 88% vs. 90.9% 2b.ii. 2.7% vs. 0% 2b.iii. 9.3% vs. 9.1% 2c. *P* = .32 2c.i. 42.7% vs. 27.3% 2c.ii. 16% vs. 59.1% 2c.iii. 41.3% vs. 13.6% 2d. *P* = .35 2d.i. 32% vs. 45.5% 2d.ii. 5.3% vs. 9.1% 2d.iii. 62.7% vs. 45.5% 2e. *P* = .96 2e.i. 20% vs.22.7% 2e.ii.38.7% vs.36.3% 2e.iii. 41.3% vs.40.9% | 0 | 0 |
| Burack 2003[24] (USA) | 4 Pre-specified “primary” outcomes listed. **1. Primary care visit during study year; %; adjusted OR (95% CI).  2. Mammogram completed during study year; %; adjusted OR (95% CI).  3. Pap smear test completed during study year; %; adjusted OR (95% CI).**  Subgroup analyses (not prespecified). 4. In women who had a mammogram < 2 years before study. 4a. Primary care visit in study year.  4b. Mammogram completed during study year.  4c. Pap smear test completed during study year.  5. In women who had a pap smear test in < 2 years before study (%).  5a. Primary care visit in study year.  5b. Mammogram completed during study year.  5c. Pap smear test completed during study year.  No differences were reported in subgroups of women who did not have mammogram or pap smear tests in 2 years before study. | 1. 76% vs. 77%; 0.90 (0.73 to 1.11)  2. 39% vs. 40%; 0.94 (0.78 to 1.14)  3. 30% vs. 23%; 1.39 (1.07 to 1.89) 4a. 86% vs. 88%, *P* = NS 4b. 51% vs. 57%, *P* = .04 4c. 45% vs. 37%, *P* = .012 5a. 85% vs. 92%, *P* = .002 5b. 47% vs. 50%, *P* = NS 5c. 52% vs. 45%, *P* = NS | ... | ... | 0 | … |
| Burack 1998[23] (USA) | (Combined patient & physician intervention vs. physician only vs. patient only vs. control)   **1. Number (%) of patients with primary care visit: OR (compared with control): (95% CI). (primary)  2. Proportion of patients with Pap smear completed: OR, 95% CI. (primary)**  (note re #3 and #4 - these are secondary outcomes, although the breakdown by subgroups was not specified)  3. Proportion of patients with Pap smear completed at (other sub-group comparisons at each site available): Physician reminders vs. no physician reminders a. site 1 b. site 2 c. site 3  4. Median time (weeks) between reminder intervention and visit (95% CI): Patient reminders vs. no patient reminders 4a. among women with a chronic illness  4b. among women without a chronic illness | 1. 960 (79%), 1.23 (0.99 to 1.52) vs. 960 (77%), 1.07 (0.87 to 1.32) vs. 964 (75%), 0.98 (0.80 to 1.21) vs. 964 (75%) reference (n/a), *P* >.05  2. 32%, 1.23 (1.01 to 1.50) vs. 29%, 1.05 (0.86 to 1.28) vs. 29%, 1.07 (0.88 to 1.30) vs. 28% reference (n/a), *P* >.05  3a. 46% vs. 44% 3b. 46% vs. 44% 3c. 44% vs. 41%  4a. no difference between groups 4b. 16 vs. 9 (adjusted coefficient 0.77, 0.13 to 1.41) | ... | ... | 0 | … |
| Burack 1997[22] (USA) | Note: This is a follow-up study to the 1994 publication and includes some patients from the 1994 study.  Prespecified **1. Mammography completion rates in study year 1. % (estimated from figure 2); adjusted OR (95% CI). Note: additional data for year 1 analyses are reported in the 1994 paper and differ slightly because N’s differ.  1a. Health Departments.  1b. HMO.  2. Mammography completion rates in study year 2. %; adjusted OR (95% CI).  2a. Health Departments.  2b. HMO.** Not prespecified  3. Difference between year 1 and year 2 effectiveness for both groups. | 1a. 58% vs. 36%; 2.74 (2.17 to 3.46) 1b. 58% vs. 47%; 1.59 (1.23 to 2.05) 2a. 44% vs. 28%; 1.85 (1.41 to 2.41) 2b. 45% vs. 46%; 1.07 (0.80 to 1.42) 3. *P* = .010 | ... | ... | + | … |
| Burack 1996[21] (USA) | 3 intervention groups (physician reminder, patient reminder, and both reminders) and 1 control group. Data reported separately for the 2 participating sites.  **Prespecified 1. Primary care visit during study year for 1527 women due for mammography within 1st 4 months of study. 1a. Site 1.  1b. Site 2.**  2. Time to 1st primary care visit after patient reminder for 1099 women due for mammography within 1st 4 months of study and continuing in HMO (median, weeks).  2a. Site 1. Patient reminder vs. no patient reminder.  2b. Site 2.  Reported by nonrandomized insurance subgroups only.  **3. Mammography rate during the study year for 1527 women due for mammography within 1st 4 months of study. 3a. Site 1.  3b. Site 2. Both physician reminder groups vs. no reminder. 3c. Site 2. Patient reminder vs. no reminder.**  4. Mammography rate for 1627 women who visited physicians during the study year. Physician reminders vs. no physician reminders. 4a. Site 1. %; OR (95% CI).  4b. Site 2. %.  Paper also reports subgroup analyses (not pre-specified) by age and due date for mammography (≤4 months, >4 months). | 1a. 63-64%, *P* = .934 (multivariate analysis) across groups. 1b. 50-59%, *P* = .466 (multivariate analysis) across groups.  2a. 9 vs. 9, *P* = .504 2b. NR  3a. Approximately 30% for each group, *P* = .524 across groups. 3b. 36%/36% vs. 22%, *P* = .002 (multivariate analysis).  3c. 21% vs. 22%, *P* = NS 4a. 48% vs. 46%; 1.01 (0.77 to 1.31). 4b. 59% vs. 43%, *P* < .001 | ... | ... | 0 | … |
| Burack 1994[20] (USA) | Prespecified **1. Evaluation of medical record reminder. Proportion of women with scheduled mammography appointments over 6 months; difference (95% CI)**  1a. Across all 5 sites.  **1b. Health Department #1.  1c. Health Department #2.  1d. Health Maintenance Organization (HMO).  1e. Hospital #1.  1f. Hospital #2.**  1g. Increase with intervention, Health Departments vs. HMO. 1h. Increase with intervention, HMO vs. hospitals  2. Evaluation of patient postcard reminder. Proportion of women with completed mammography appointment within 2 months of 1st scheduled appointments; difference (95% CI).  2a. Across all 5 sites.  2b. Health Department #1.  2c. Health Department #2.  2d. HMO.  2e. Hospital #1.  2f. Hospital #2.  3. Evaluation of rescheduling system. Proportion of women eligible for rescheduling and subsequently completing mammography appointment; difference (95% CI)  3a. Across all 5 sites.  3b. Health Department #1.  3c. Health Department #2.  3d. HMO.  3e. Hospital #1.  3f. Hospital #2.  4. Proportion of women with completed mammography appointment over 6 months (includes initial completion, deferred completion, and completion after telephone follow-up).  4a. Across all 5 sites.  4b. Health Department #1.  4c. Health Department #2.  4d. HMO.  4e. Hospital #1.  4f. Hospital #2.  **5. Evaluation of full intervention over 12 months. Proportion of women having mammography; difference (95% CI).** 5a. Across all 5 sites.  **5b. Health Department #1.  5c. Health Department #2.  5d. HMO.  5e. Hospital #1.  5f. Hospital #2.**  Note: The overall outcome was not evaluated for outcomes 1 and 5 because no statistical comparison was reported. | 1a. 47% vs. 25%; NR 1b. 65% vs. 37%; 28.7% (20.7 to 36.7) 1c. 40% vs. 11%; 29.3% (21.1 to 37.5) 1d. 41% vs. 28%; 13% (5.9 to 20) 1e. 38% vs. 23%; 15.3% (5.2 to 25.4) 1f. 46% vs. 24%; 22.7% (14.8 to 30.6)  1g. 29% vs. 13%, *P* = .005 1h. 13% vs. 19%, *P* = .202 2a. 77% vs. 78%; NR 2b. 77% vs. 84%; -6.6% (-16.1 to 2.9) 2c. 83% vs. 57%; 25.5% (2.7 to 48.4) 2d. 82% vs. 80%; 1.3% (-8.9 to 11.5) 2e. 81% vs. 74%; 7.1% (-10.5 to 24.6) 2f. 67% vs. 74%; -6.3% (-20.1 to 7.4)  3a. 23% vs. 22%; NR 3b. 32% vs. 13%; 19.2% (-2.4 to 40.8) 3c. 38% vs. 22%; 16.2% (-21.7 to 54.1) 3d. 37% vs. 16%; 21.2% (-3.3 to 45.8) 3e. 36% vs. 22%; 14.1% (-25.2 to 53.5) 3f. 37% vs. 69%; -32.2% (-59.2 to -5.1)  4a. 85% vs. 84%, *P* = NS 4b. 84% vs. 86%, *P* = NS 4c. 89% vs. 67%, *P* = NS 4d. 88% vs. 83%, *P* = NS  4e. 88% vs. 80%, *P* = NS 4f. 79% vs. 92%, *P* = NS 5a. 53% vs. 41%; NR 5b. 64% vs. 44%; 19.5% (11.6 to 27.5) 5c. 50% vs. 25%; 25.2% (16.3 to 34.2) 5d. 59% vs. 46%; 12.1% (5.2 to 19.1) 5e. 43% vs. 28%; 14.2% (4.0 to 24.4) 5f. 45% vs. 28%; 16.5% (9.0 to 24.0) | ... | ... | + | … |
| McPhee 1991[40] (USA) | Prespecified. 1. Compliance with American Cancer Society and/or National Cancer Institute recommendations over 12 months (Mean %, SD). 1a. FOBT.  1b. Rectal exam.  1c. Sigmoidoscopy.  1d. Pap smear test (>100% means test done more frequently than recommended).  1e. Pelvic exam.  1f. Breast exam.  1g. Mammography.  1h. Smoking assessment.  1i. Smoking counselling.  1j. Diet assessment.  1k. Diet counselling.   **2. Multiple regression analysis for effect of reminders over 12 months, controlling for preintervention activity scores (unstandardised regression coefficient β, p-value). 2a. FOBT.  2b. Rectal exam.  2c. Sigmoidoscopy.  2d. Pap smear test.  2e. Pelvic exam.  2f. Breast exam.  2g. Mammography.  2h. Smoking assessment.  2i. Smoking counselling.  2j. Diet assessment.  2k. Diet counselling.** | 1a. 50.4% (17.3) vs. 34.2% (13.0), *P* = .002 1b. 49.6% (15.7) vs. 40.3% (12.4), *P* = .047 1c. 39.5% (41.9) vs. 31.4% (27.1), *P* = .480 1d. 154.7% (44.8) vs. 120.9% (48.4), *P* = .029 1e. 54.8% (14.1) vs. 41.4% (14.4), *P* = .006 1f. 57.3% (17.6) vs. 48.7% (15.8), *P* = .118 1g. 40.1% (14.2) vs. 34.9% (13.7), *P* = .245 1h. 45% (16.6) vs. 32.4% (13.9), *P* = .014 1i. 58.8% (23.0) vs. 41.8% (22.2), *P* = .027  1j. 23% (23.8) vs. 7% (11.4), *P* = .011  1k. 17.5% (14.0) vs. 0.6% (1.4), *P* = .003 2a. 14.5, *P* = .001  2b. 10.5, *P* = .004  2c. 11.4, *P* = .270 2d. 30.7, *P* = .014 2e. 11.8, *P* = .002  2f. 8.7, *P* = .032 2g. 4.7, *P* = .26 2h. 10.2, *P* = .021  2i. 17.3, *P* = .027  2j. 12.3, *P* = .011  2k. 13.9, *P* = .001  All in favour of CCDSS. | ... | ... | + | … |
| McPhee 1989[39] (USA) | Prespecified. **CCDSS vs. control;** audit + feedback vs. control 1. Compliance with American Cancer Society recommendations over 9 month intervention (Mean compliance score shown in figure 2 of article but data not provided; only p-value for the comparison is provided). 1a. FOBT.  1b. Rectal exam.  1c. Sigmoidoscopy.  1d. Pap smear test.  1e. Pelvic exam.  1f. Breast exam.  1g. Mammography.   2. Multiple regression analysis for compliance over 9 months, controlling for preintervention compliance (unstandardised regression coefficient β, p-value). 2a. FOBT.  2b. Rectal exam.  2c. Sigmoidoscopy.  2d. Pap smear test.  2e. Pelvic exam.  2f. Breast exam.  2g. Mammography.   **3. Multiple regression analysis for provider performance over 9 months, controlling for physician case mix (unstandardised regression coefficient β, p-value).  3a. FOBT.  3b. Rectal exam.  3c. Sigmoidoscopy.  3d. Pap smear test.  3e. Pelvic exam.  3f. Breast exam.  3g. Mammography.**   Note: Data for patient reminders are not reported here because they were not presented by CCDSS vs. no CCDSS | For outcomes 1-3, significant p-values are all in favour of CCDSS (i.e. compliance higher with CCDSS).  1a. *P* < .01; *P* = NS 1b. *P* < .001; *P* = NS 1c. *P* < .01; *P* = NS 1d. *P* = NS; *P* = NS  1e. *P* < .01; *P* = NS 1f. *P* < .01; *P* < .01  1g. *P* < .05; *P* < .01  2a. 19.0, *P* = .002; 12.3, *P* = .048 2b. 22.6, *P* < .001; 14.0, *P* = .02 2c. 31.3, *P* = .002; -1.2, *P* = .899 2d. 34.8, *P* = .122; 29.5, *P* = .198 2e. 20.5, *P* = .004; 10.4, *P* = .140 2f. 24.3, *P* = .001; 25.3, *P* = .001 2g. 15.7, *P* = .04; 20.6, *P* = .008 3a. 14.8, *P* = .002; 6.7, *P* = .148 3b. 17.6, *P* < .001; 8.0, *P* = .059 3c. 6.9, *P* = .002; 0.5, *P* = .813 3d. 8.5, *P* = .112; 5.1, *P* = .353 3e. 14.8, *P* = .003; 6.5, *P* = .195 3f. 18.5, *P* < .001; 14.1, *P* = .006 3g. 11.0, *P* = .031; 10.0, *P* = .05 | ... | ... | + | … |
| **Multiple preventive care activities** | | | | | | |
| Harari 2008[34] (England, UK) | **All outcomes reported as % (numbers) of self-reported behavior/uptake of patients, intervention vs. control, OR (95% CI); p-value at 1 year follow-up. Article calls all of these outcomes primary. 1. Blood-pressure check in previous year  2. Cholesterol measurement in previous 5 years (younger than 75 years)  3. Blood glucose measurement in previous 3 years  4. FOBT in previous year (younger than 80 years)  5. Influenza vaccination in previous year  6. Pneumococcal vaccination (ever)  7. Dental check in previous year  8. Vision check-up in previous year 9. Hearing check-up in previous year  10. Mammography in previous 2 years (younger than 70 years)** | 1. 83.5% (785/940) vs. 84.8% (903/1066), 0.9 (0.7 to 1.2); *P* = .40 2. 60.2% (312/518) vs. 60.4% (389/643), 1.0 (0.8 to 1.3); *P* = .95 3. 25.9% (243/940) vs. 27.2% (302/1066), 0.9 (0.7 to 1.1); *P* = .19 4. 6.1% (45/732) vs. 5.7% (49/862), 1.1 (0.7 to 1.6); *P* = .73 5. 83.9% (788/939) vs. 85.8% (916/1066), 0.8 (0.6 to 1.1); *P* = .12 6. 32.8% (308/939) vs. 27.5% (291/1066), 1.2 (1.01 to 1.5); *P* = .04 7. 74.9% (678/905) vs. 72.0% (757/1051), 1.1 (0.9 to 1.4); *P* = .23 8. 68.3% (626/916) vs. 69.6% (732/1052), 0.9 (0.8 to 1.1); *P* = .53 9. 17.0% (155/912) vs. 18.2% (191/1047), 0.9 (0.7 to 1.2); *P* =.47 10. 35.9% (47/131) vs. 32.3% (50/155), 1.2 (0.7 to 1.9); *P* = .52 | **All outcomes reported as % (numbers) of self-reported behavior/uptake of patients, intervention vs. control, OR (95% CI); p-value at 1 year follow-up. Article calls all of these outcomes primary. 1. ≥3 times per week moderate or strenuous physical activity  2. ≥5 times per week moderate or strenuous physical activity  3. Consumption of ≤2 high fat food items per day  4. Consumption of ≥5 fruit/fibre items per day**  **5. No current tobacco use  6. No or moderate alcohol use  7. Driving with use of seat belt** | 1. 16.4% (143/874) vs. 13.8% (137/993), 1.2 (0.9 to 1.6); *P* = .15 2. 10.8% (94/872) vs. 7.8% (77/989), 1.4 (1.0 to 2.0); *P* = .03 3. 25.2% (219/870) vs. 21.8% (218/999), 1.2 (0.95 to 1.5); *P* = .13 4. 37.2% (326/877) vs. 36.7% (372/1015), 1.0 (0.8 to 1.3); *P* = .86 5. 90.9% (779/857) vs. 89.6% (897/1001), 1.2 (0.9 to 1.6); *P* = .36 6. 80.2% (727/906) vs. 79.7% (822/1032), 1.1 (0.8 to 1.3); *P* = .63 7. 84.1% (755/898) vs. 84.9% (883/1040), 1.0 (0.7 to 1.2); *P* = .66 | 0 | 0 |
| Apkon 2005[16] (USA) | Primary (and components of primary) 1. Healthcare opportunities fulfilled at 60 days, n (%); OR (95% CI).  **2. Screening/prevention healthcare opportunities fulfilled at 60 days n (%).  2a. Overall.** 2b. Alcohol screening.  2c. Breast cancer.  2d. Cervical cancer.  2e. Chlamydia.  2f. Colorectal cancer.  2g. Depression.  2h. Dietary counselling.  2i. Exercise counselling.  2j. Lipid.  2k. Pneumococcal vaccine.  2l. Smoking/advice to quit.  2m. Smoking screening.  3. Acute/chronic healthcare opportunities fulfilled at 60 days n (%).  **3a. Lipid abnormalities.**  **Secondary 4. Mean patient satisfaction score at 60 days (scale range and anchors not described). 4a. Speed, efficiency, and courtesy during visit.  4b. Health care provider.  4c. Personal issues.  4d. Overall visit assessment.**  Data also reported separately in article for the 2 participating sites. | 1. 805/2374 vs. 695/2265 (33.9% vs. 30.7%), *P* = .12; OR 1.14 (0.95 to 1.38), *P* = .16 2a. 722/2074 vs. 603/1983 (34.8% vs. 30.4%), *P* = .03 2b. 51/79 vs. 36/68 (64.6% vs. 52.9%), *P* = .07 2c. 3/11 vs. 4/12 (27.3% vs. 33.3%), *P* = .43 2d. 26/95 vs. 22/98 (27.4% vs. 22.4%), *P* = .47 2e. 22/73 vs. 19/64 (30.1% vs. 29.7%), *P* = .90 2f. 4/32 vs. 2/58 (12.5% vs. 3.4%), *P* = .15 2g. 164/422 vs. 155/419 (38.9% vs. 37.0%), *P* = .58 2h. 149/493 vs. 108/449 (30.2% vs. 24.1%), *P* = .04 2i. 157/509 vs. 109/462 (30.8% vs. 23.6%), *P* = .01 2j. 13/49 vs. 18/48 (26.5% vs. 37.5%), *P* = .32 2k. 1/61 vs. 0/72 (1.6% vs. 0%), *P* = .25 2l. 92/209 vs. 101/200 (44.0% vs. 50.5%), *P* = .14 2m. 40/41 vs. 29/33 (97.6% vs. 87.9%), *P* = .08  3a. 12/66 vs. 11/69 (18.2% vs. 15.9%), *P* = .81  4a. 4.17 vs. 4.19, *P* = .23 4b. 4.40 vs. 4.37, *P* = .82 4c. 4.24 vs. 4.27, *P* = Not applicable 4d. 4.27 vs. 4.30, *P* = .74 | **Not prespecified 1. Adverse events.** | 1. None reported. | 0 | 0 |
| Dexter 2001[29] (USA) | (primary outcomes-"the rates at which the various preventive therapies were ordered")  **1 Proportion of hospitalizations with an order for therapy 1a. Pneumococcal vaccination 1b. Influenza vaccination 1c. Prophylactic heparin 1d. Prophylactic aspirin at discharge  2 Proportion of hospitalizations during which therapy was ordered for an eligible patient 2a. Pneumococcal vaccination 2b. Influenza vaccination 2c. Prophylactic heparin 2d. Prophylactic aspirin at discharge** | 1a. 8.5% vs. 0.9%, *P* < .001 1b. 5.4% vs. 0.4%, *P* < .001 1c. 10.5% vs. 8.2%, *P* < .001 1d. 29.7% vs. 25.4%, *P* = .005  2a. 35.8% vs. 0.8%, *P* < .001 2b. 51.4% vs. 1.0%, *P* < .001 2c. 32.2% vs. 18.9%, *P* < .001 2d. 36.4% vs. 27.6%, *P* < .001 | ... | ... | + | … |
| Demakis  2000[28] (USA) | **Primary outcomes 1. Proportion of patients in compliance with all 13 standards of care over 17 months. N, % adherent; OR (95% CI). 1a. Hypertension: weight, exercise, sodium. 1b. Diabetes: nutrition counselling. 1c. Age ≥65 years or high risk: pneumonoccal vaccination. 2. Proportion of all visits for which care was indicated and residents provided proper care over 17 months. N, % adherent; OR (95% CI). 2a. Hypertension: weight, exercise, sodium. 2b. Diabetes: nutrition counselling. 2c. Age ≥65 years or high risk: pneumococcal vaccination.** | 1a. 4244, 55.2% vs. 4471, 49.3%; 1.27 (0.92 to 1.75), *P* = .14  1b. 1896, 61.6% vs. 2064, 53.3%; 1.29 (0.93 to 1.79), *P* = .12  1c. 1759, 12.7% vs. 1688, 4.3%; 3.26 (2.09 to 5.09), *P* < .001  2a. 3540, 17.0% vs. 3896, 10.3%; 1.77 (1.53 to 2.05), *P* < .001  2b. 1596, 17.0% vs. 1800, 13.7%; 1.29 (1.05 to 1.58), *P* = .02  2c. 883, 7.9% vs. 829, 1.1%; 7.85 (3.83 to 16.08), *P* < .001 | ... | ... | + | … |
| Overhage 1996[42] (USA) | **Primary outcomes 1. Compliance with preventive care guidelines over 6 months: Number of eligible patients (% compliance).** 1a. Overall. **1b. Cervical cytology study. 1c. Pneumococcal vaccination. 1d. Aspirin. 1e. Estrogen treatment. 1f. Calcium treatment. 1g. Opthalmologic referral. 1h. Mammography. 1i. Thyroid-stimulating hormone screen. 1j. Hepatitis B screen. 1k. Rubella screen. 1l. Screening urinalysis. 1m. Cholesterol test. 1n. Pregnancy test. 1o. HIV screen.**  **1p. Heparin prophylaxis. 1q. 24h urine protein screen. 1r. Sickle cell screen. 1s. Screening electrocardiogram. 1t. Sexually transmitted disease screen. 2. Attitude towards providing preventive care to hospitalised patients at 6 months (prespecified).** | 1a. 23% vs. 24%, *P* = .78 1b. 323 (2.8%) vs. 329 (2.8%), *P* = .41  1c. 271 (2.6%) vs. 243 (2.1%), *P* = .69 1d. 246 (9.4%) vs. 247 (9.7%), *P* = .89 1e. 243 (0.8%) vs. 232 0.3%), *P* = .62 1f. 243 (5.4%) vs. 232 (3.9%), *P* = .45 1g. 217 (2.3%) vs. 200 (1.5%), *P* = .55 1h. 125 (5.6%) vs. 131 (1.5%), *P* = .08 1i. 112 (16.1%) vs. 118 (9.3%), *P* = .12 1j. 88 (8.0%) vs. 92 (2.2%), *P* = .08 1k. 80 (1.2%) vs. 86 (0.3%), *P* = .30 1l. 68 (32.4%) vs. 75 (34.7%), *P* = .77 1m. 70 (14.3%) vs. 58 (13.8%), *P* = .94 1n. 60 (13.3%) vs. 66 (13.6%), *P* = .96  1o. 44 (4.6%) vs. 43 (9.3%), *P* = .38 1p. 30 (43.3%) vs. 28 (35.7%), *P* = .55 1q. 24 (25.0%) vs. 23 (4.4%), *P* = .05  1r. 22 (9.0%) vs. 14 (0%), *P* = .25 1s. 13 (0%) vs. 14 (21.4%), *P* = .08  1t. 2 (50%) vs. 6 (16.7%), *P* = .35 2. No difference (data not reported) | ... | ... | 0 | … |
| Frame 1994[33] (USA) | **Prespecified 1. Overall change in provider compliance with 11 health maintenance procedures over 2 years (%). 1a. For 1,324 initially active patients (those seen ≥ 1 time in previous 2 years).  1b. For 145 initially inactive patients.** 2. Change in provider compliance with 11 specific health maintenance procedures over 2 years for initially active or inactive patients: total N (% initial compliance); % change in compliance; difference (95% CI). 2a. Teach self-exam.  2b. Teach to report postmenopausal bleeding.  2c. Mammography.  2d. Tetanus booster.  2e. FOBT.  2f. Clinical breast exam.  2g. Papanicolaou test.  2h. Cholesterol measurement.  2i. BP measurement.  2j. Weight measurement.  2k. History of tobacco use.   Not prespecified 3. Proportion of patients active at final audit.  3a. Initially inactive patients (n=145).  3b. All patients.  3c. Initially active patients (those seen at least once in previous 2 years, n=1,324).   Note: study included inactive and never seen patients only if a family member was active | 1a. 13.5% vs. 3.3%, *P* < .001  1b. 27.1% vs. 13.5%, *P* = .02 2a. 1469 (4% vs. 3%); 37% vs. 10%, 27% (23 to 31)  2b. 261 (9% vs. 10%); 39% vs. 13%; 26% (15 to 35) 2c. 261 (44% vs. 47%); 11% vs. -12%; 23% (9 to 40) 2d. 1469 (20% vs. 21%); 36% vs. 15%; 21% (16 to 26) 2e. 776 (40% vs. 34%); 18% vs. 3%; 15% (6 to 23) 2f. 806 (49% to 47%); 8% vs. -3%; 11% (2 to 19) 2g. 696 (52% vs. 52%) ; 10% vs. 1%; 9% (1 to 19) 2h. 1268 (48% vs. 45%); 17% vs. 11%; 6% (1 to 11) 2i. 1469 (89% vs. 86%); -7% vs. -10%; 3% (-2 to 7) 2j. 1469 (93% vs. 94%); 1% vs. -1%; 2% (-1 to 4) 2k. 1469 (82% vs. 80%); 11% vs. 11%; 0% (-3 to 4) 3a. 51% vs. 37%, *P* = .81 3b. 73% vs. 69%, *P* = .059 3c. 82% vs. 78%, *P* = .045 | ... | ... | + | … |
| Turner 1994[53] (USA) | Primary outcome **1. % performance of health maintenance activities over 1 year: Baseline/follow-up; difference.  1a. Influenza vaccinations. 1b. Stool for occult blood test. 1c. Pap smears. 1d. Breast examinations performed by physicians. 1e. mammograms.** | 1a. 20%/26% vs. 17%/24%; +6% vs. +7%, *P* = .51 1b. 30%/31% vs. 28%/23%; +1% vs. -5%, *P* = .70 1c. 23%/26% vs. 26%/15%; +3% vs. -11%, *P* = .10 1d. 30%/33% vs. 35%/33%; +3% vs. -2%, *P* = .64  1e. 15%/26% vs. 22%/25%, +11% vs. +3%, *P* = .41 | ... | ... | 0 | … |
| Ornstein 1991[41] (USA) | **1. Change (95% CI) in proportion of patients who received each of the five preventive services over 1 year (prespecified). Physician reminders vs. patient reminders vs. both vs. neither (control).  1a. Cholesterol measurement. 1b. FOBT. 1c. Mammography. 1d. Papanicolaou smear. 1e. Tetanus vaccine.**  ** data also available by quarter | 1a. 12.3% (11.3 to 13.2) vs. 13.6% (13.0 to 14.3) vs. 18.6% (17.8 to 19.5) vs. 9.1% (8.0 to 10.1) Combined reminder group showed significantly greater improvement than other groups by pairwise comparisons.  1b. 5.1% (1.8 to 8.5) vs. 8.7% (5.8 to 11.6) vs. 17.7% (14.9 to 20.4) vs. 8.1% (4.7 to 11.5)  Combined reminder group showed significantly greater improvement than other groups by pairwise comparisons.  1c. 10.7% (4.7 to 16.8) vs. 2.8% (-3.0 to 8.5) vs. 15.7% (11.1 to 20.2) vs. 15.7% (10.7 to 20.9)  1d. -4.5% (-7.1 to -1.9) vs. -2.1% (-4.7 to 0.5) vs. -0.8% (-3.7 to 2.1) vs. -0.9% (-4.0 to 2.1) 1e. 10.5% (9.8 to 11.3) vs. 9.5% (8.9 to 10.1) vs. 12.0% (11.2 to 12.8) vs. 3.8% (3.1 to 4.4) Each of the 3 intervention groups showed significantly greater improvements than the control group. | ... | ... | + for combined reminder group  0 for physician and patient reminder groups | … |
| Rosser 1991[46] (Canada) | **1. (prespecified) Percentage of patients for whom the recommended procedure was performed (physician reminder, letter reminder, telephone reminder, control).** 1a. Influenza vaccine 1b. BP measurement 1c. Smoking status assessed 1d. Pap smear 1e. Tetanus vaccine 1f. male, 15-34 years 1g. male, 35-64 years 1h. male, ≥65 years 1i. male, all 1j. female, 15-34 years 1k. female, 35-64 years 1l. female, ≥65 years 1m. female, all  **1n. overall** 1o. male 15-44 years 1p. male ≥45 years 1q. female 15-64 years  1r. female ≥ 65 years | 1 (physician reminder, letter reminder, telephone reminder, control) 1a. 22.9, 35.2, 37.0, 9.8 (p-value not indicated) 1b. 30.7, 40.5, 37.2, 21.1 (p-value not indicated) 1c. 37.9, 49.4, 55.8, 11.9 (p-value not indicated) 1d. 16.5, 29.7, 30.0, 13.7 (p-value not indicated) 1e. 22.8, 30.6, 24.0, 3.2 (p-value not indicated) 1f. 20.5*, 31.3*†, 37.7*†‡, 8.3 1g. 34.7, 49.4*†, 43.4*†‡, 14.9 1h. 44.7, 52.1*†, 43.0*†‡, 13.7 1i. 30.3*, 43.0*†, 41.2*†‡, 12.3 1j 26.7*, 35.8*†, 39.9*, 13.6 1k. 38.8*, 45.8*†, 45.1*†, 14.5 1l. 38.4, 47.1*†, 42.7*†‡, 10.7 1m. 33.7*, 42.0*†, 42.0*†, 13.5 * Significantly greater than proportion in control group (*P* < .01) †Significantly greater than proportion in physician reminder group (*P* < .05) ‡Significantly different from proportion in letter reminder group (*P* < .05)  1n. 33.3, 42.0, 42.0, 14.1%, *P* < .05 for telephone or letter reminder vs. physician reminder; *P* < .001 for intervention groups vs. control.  1o. telephone reminder more effective than letter (*P* < .05) 1p. letter reminder more effective than telephone reminder (*P* < .05) 1q. letter similarly effective to telephone reminder  1r. letter reminder more effective than telephone reminder (*P* < .05) | ... | ... | + | … |
| Tierney 1986[52] (USA) | Primary outcomes **1. Percent physician compliance with Group A and Group B preventive care protocols over 7 months (4 groups: monthly feedback + reminders at patient visit vs. monthly feedback only vs. reminders only vs. no feedback or reminders (control)).** (Limited data reported.)  Group A Protocols: **1a. Fecal blood testing (N=2991). 1b. Pneumococcal vaccination (N=1759). 1c. Antacids (N=1343). 1d. TB skin testing (N=1383).**  Group B Protocols: **1e. Calcium supplements (N=2713). 1f. Cervical cytology (N=1636). 1g. Mammography (N=1539). 1h. Salicylates (N=97).** | 1a. *P* < .01 in favour of monthly feedback or reminders vs. control*  1b. *P* < .01 in favour of monthly feedback or reminders vs. control*  1c. *P* = NS across all groups.  1d. *P* = NS across all groups. 1e. *P* < .01 in favour of reminders vs. control, regardless of monthly feedback 1f. *P* < 0.05 in favour of control vs. reminders, regardless of monthly feedback  1g. *P* < 0.01 in favour of monthly feedback or reminders vs. control* 1h. *P* = NS across all groups.   *Effects of monthly feedback and reminders were not additive. | ... | ... | + | … |
|  | **Screening and management of CV risk factors** |  |  |  |  |  |
| Bertoni 2009[18, 19] (USA) | 3-year follow-up 1. Lipid screening rates for patients (secondary)  1a. Proportion of patients at baseline (N=2216 vs. 2841); Difference 1b. Proportion of patients at follow-up (N=1811 vs. 2010); Difference 1c. Change from follow-up to baseline; Difference; intra-class correlation  **2. Appropriate lipid management (met 1 of 7 criteria based on LDL-C level and risk strata) (primary)** 2a. Proportion of patients at baseline (N=842 vs. 855); Difference 2b. Proportion of patients at follow-up (N=709 vs. 771); Difference **2c. Change from follow-up to baseline; Difference; intra-class correlation** 2d. Group difference in subgroup of 58 practices with both baseline and follow-up data. Not prespecified   3. Inappropriate prescription of lipid-lowering therapy (secondary). 3a. Proportion of patients at baseline (N=626 vs. 650); Difference 3b. Proportion of patients at follow-up (N=519 vs. 571); Difference 3c. Change from follow-up to baseline; Difference  4. Appropriate prescription of lipid-lowering therapy. (secondary) 4a. Proportion of patients at baseline (N=216 vs. 205); Difference 4b. Proportion of patients at follow-up (N=190 vs. 200); Difference 4c. Change from follow-up to baseline; Difference  Stratified subgroup analyses 5-7. Appropriate lipid management of patient dyslipidemia by Risk Category* 5. Low risk patients: baseline N=296 vs. 357; follow-up N=309 vs. 336 6. Intermediate low-risk or intermediate high-risk: baseline N=315 vs. 281; follow-up N=253 vs. 254 7. High risk patients: baseline N=231 vs. 217; follow-up N=147 vs. 181 a. Proportion of patients at baseline; Difference. b. Proportion of patients at follow-up; Difference c. Change from follow-up to baseline; Difference; intraclass correlation  *Risk category defined by Framingham risk score (history and 10-year risk of CHD)  (1) Low risk (0-1 risk factors for CHD); (2) intermediate low risk (≥2 risk factors and a 10-year risk of <10%) (3) intermediate high risk (≥2 risk factors and a 10-year risk of 10% to 20% ) (4) high risk (CHD risk equivalent [diabetes, CHD, stroke, or peripheral vascular disease] and/or ≥2 risk factors with a 10-year risk of >20%) | 1a. 43.6% vs. 40.1%; +3.5; *P* = .41 1b. 49% vs. 50.8%; -1.8; *P* = .72 1c. +6.6 vs. +10.7; -5.3; *P* = .22; 0.22  2a. 73.4% vs. 79.7%; -6.3; *P* = .02 2b. 72.3% vs. 68.9%; +3.4; *P* = .18 2c. -1.1 vs. -10.8; +9.7; *P* < .01; 0.01 2d. +9.2%, *P* = .02  3a. 6.6% vs. 4.2%; +2.4; *P* = .15 3b. 3.9% vs. 6.4%; -2.5; *P* = .07 3c. -2.7 vs. +2.2; -4.9; *P* = .01  4a. 38.8% vs. 45.3%; -6.5; *P* = .27 4b. 24.8% vs. 24.1%; +0.7; *P* = .88 4c. -14.0 vs. -21.2; +7.2; *P* = .37  5a. 91.4% vs. 94.1%; -2.7; *P* = .29 5b. 90.9% vs. 89.2%; +1.7; *P* = .49 5c. -0.5 vs. -4.9; +4.4; *P* = .21; 0.01  6a. 69.4% vs. 73.9%; -4.5; *P* = .60 6b. 70.3% vs. 62.6%; +7.7; *P* = .07 6c. +0.9 vs. -7.3; +8.2; *P* = .03; 0.01  7a. 47.5% vs. 55.6%; -8.1; *P* = .14 7b. 24.4% vs. 28.7%; -4.3; *P* = .41 7c. -23.1 vs. -26.9; +3.8; *P* = .65; 0.01 | ... | ... | + | … |
| Van Wyk 2008[56] (The Netherlands) | **2 primary outcomes, 12-month follow-up  (auto alerting vs. on-demand vs. control) 1. Patients requiring screening who were screened.**  1a. n/N (%) patients. 1b. RR (95% CI) adjusted for individual visits and practice size **1bi. Auto alerting vs. control. 1bii. On-demand vs. control.** 1biii. Auto alerting vs. on-demand. | 1a. 701/1079 (65%) vs. 438/1249 (35.1%) vs. 225/882 (25.5%) 1bi. 1.76 (1.41 to 2.20) 1bii. 1.28 (0.98 to 1.68) 1biii. 1.40 (1.08 to 1.81) | ... | ... | Auto, +  On-demand, 0 | … |
| Unrod 2007[54, 55] (USA) | **Primary**  **(Assessed by patients after visits) intervention vs. control (%) OR (if reported); 95% CI (if reported); whether the physician 1. Asked if the patient smoked 2. Assessed the willingness to quit 3. Provided quitting advice 4. Helped the patient set** **goals 5. Provided written materials 6. Referred patient to quit-smoking program 7. Discussed quit-smoking medications 8. Arranged a follow-up appointment**  Cost-effectiveness was evaluated from perspective of individual physician practices (not prespecified in main paper).  9. Overall incremental cost-effectiveness (US $)  9a. Per life-year saved.  9b. Per quality-adjusted life-year saved.  10. Incremental cost-effectiveness per net quitter. (US $)  10a. Prepreparation stage.  10b. Preparation stage.  10c. Overall | 1. 61.2% vs. 47.4%,  2. 76% vs. 36.8%, 5.06 (3.22 to 7.95). 3. 76.8% vs. 53%, 2.79 (1.70 to 4.59). 4. 55.1% vs. 20.2%, 4.31 (2.59 to 7.16). 5. 32.3% vs. 6.9%, 5.14 (2.60 to 10.14). 6. 23.2% vs. 4.5%, 4.72 (2.90 to 7.68). 7. 61.6% vs. 24.7%, 6.48 (3.11 to 13.49). 8. 47.5% vs. 9.7%, 8.14 (3.98 to 16.68).  9a. $1,174  9b. $869  10a. $4,757  10b. $735  10c. $1,715 | **Primary at 6-month post-intervention  1. The 7-day point-prevalence abstinence for intervention vs. control, p-value.**  Secondary  2. The longest quit attempt in days M (appears to be ‘mean’ but not explicit) (SD) for intervention vs. control, p-value. 3. total number of 24-hour quit attempts M(SD) for intervention vs. control, p-value. 4. Stage-of-change-progression change score variable calculated as the difference between baseline and 6-month stage scores. | 1. 12% vs. 8%, *P*=.078 2. 18.4 (36.7) vs. 12.4 (29.6), *P*=.05 3. 2.1 (3.4) vs. 2.1 (3.5), *P*=.91 4. F=3.84, df=465, *P* < .05 in favour of CCDSS | + | 0 |
| Cobos 2005[27] (Spain) | Mean follow-up 12.2 vs. 11.2 months **All secondary**  **1. Number (%) patients treated with LLDs; OR (95% CI). 1a. High-risk patients without CHD. 1b. Low-risk patients without CHD.** | 1a. 201 (70.5%) vs. 260 (76.9%); 0.69 (0.44 to 1.06) 1b. 124 (19.0%) vs. 292 (44.2%); 0.25 (0.16 to 0.41) | Mean follow-up 12.2 vs. 11.2 months. **1. Proportion of patients with successful management (subgroup analysis not prespecified) (ITT: 1046 vs. 1145 patients).**  **1a. High-risk patients without CHD and no previous LLD treatment. 1b. High-risk patients without CHD and previous LLD treatment. 1c. Low-risk patients without CHD and no previous LLD treatment. 1d. Low-risk patients without CHD and previous LLD treatment.**  Management success: *If CV risk ≥20% over 10 years, success = LDL-C < 115mg/dL at study end for patients with CHD or < 130mg/dL for those without CHD. If CV risk <20% over 10 years, success = CV risk still <20% at study end. | 1a. 21.53% vs. 21.25%, *P* = NS 1b. 20.20% vs. 19.94%, *P* = NS 1c. 73.68% vs. 73.36%, *P* = NS 1d. 72.09% vs. 71.76%, *P* = NS Note: No significant interactions for group by CV risk level or group by previous LLD treatment. | + | 0 |
| Kenealy 2005[35] (New Zealand) | **1. The primary prespecified physician outcome was the percentage of patients (who were eligible for diabetes screening and who visited a family practitioner during the study) screened for diabetes in the computer reminder group vs. patient reminder group vs. group with both patient & computer reminders vs. control group (usual care) over two months; OR (95% CI) patient reminders vs. usual care; OR (95% CI) computer reminders vs. usual care; OR (95% CI) both reminders vs. usual care;** OR (95% CI) computer reminders vs. patient reminders.  2. ORs for eligible patients being screened over two months, OR, standard error, z, p>/z/, 95% confidence interval for a. both reminders  b. computer reminders c. patient reminders d. Family practitioner, female e. Family practitioner, number in practice f. Family practitioner, year graduation g. Family practitioner, tenths worked h. Family practitioner, mean number patients per day i. Family practitioner, proportion patients age 50+ years j. Family practitioner, prior screen rate k. Family practitioner, fee to patient l. Patient ,age m. Patient, “regular” n. Patient, number of visits o. Patient, non-European ethnicity | 1. 31.8 % vs. 23.9% vs. 23.7% vs. 15.5%; 1.72 (1.21 to 2.43); 2.55 (1.68 to 3.88); 1.69 (1.11 to 2.59); 1.49 (1.07 to 2.07). 2a. 1.86, 0.39, 2.94, *P* = .003, 1.23 to 2.82 2b. 2.66, 0.72, 3.63, *P* < .001, 1.57 to 4.53 2c. 1.95, 0.37, 3.53, *P* < .001, 1.35 to 2.80 2d. 1.12, 0.17, 0.72, *P* = .47, 0.83 to 1.50 2e. 0.97, 0.02, -1.35, *P* = .18, 0.94 to 1.01 2f. 1.01, 0.01, 1.35, *P* = .18, 0.99 to 1.03 2g. 0.98, 0.03, -0.54, *P* = .59, 0.92 to 1.05 2h. 1.00, 0.001, -0.59, *P* = .56, 1.00 to 1.00 2i. 27.3, 1.83, 1.53, *P* = .13, 0.75 to 10.14 2j. 4.38, 2.46, 2.63, *P* = .01, 1.46 to 13.18 2k. 1.00, 0.01, -0.01, *P* = .99, 0.98 to 1.02 2l. 1.00, 0.004, -0.82, *P* = .41, 0.99 to 1.00 2m. 2.04, 0.27, 5.44, *P* < .001, 1.58 to 2.64 2n. 1.23, 0.05, 5.44, *P* = <.001, 1.14 to 1.32 2o. 1.45, 0.20, 2.70, *P* = .007, 1.11 to 1.89 | ... | ... | + | … |
| Filippi 2003[31] (Italy) | **1. N (%) patients with antiplatelet drug prescription: baseline (12 month pre-study/follow-up (over 7 month study); difference (%); OR (95% CI). 1a. Patients with 1 cardiac risk factor and without CVD. (N=2,651 vs. 2,578) 1b. Patients with ≥ 2 cardiac risk factors and without CVD. (N=1,577 vs. 1,440)** | 1a. 358 (13.5%)/736 (27.8%) vs. 263 (10.2%)/440 (17.1%); 378 (14.3%) vs. 177 (6.9%); 2.38 (1.97 to 2.87)  1b. 224 (14.2%)/508 (32.2%) vs. 180 (12.5%)/276 (19.2%); 284 (18.0%) vs. 9.6 (6.7%); 3.22 (2.52 to 4.12) | ... | ... | + | … |
| Lowensteyn 1998[38] (Canada) | Main outcome (not specified as primary) **1. Ratio for high-risk/low-risk patients returning for reassessment at 3 months; intervention group (95% CI) vs. control group (95% CI), difference (95% CI).** | 1. 1.23 (0.96 to 1.60) vs. 0.77 (0.58 to 1.03), 0.46 (0.08 to 0.87). | Prespecified: Intervention (Profile) group vs. Control **1. Mean (SD) change in total cholesterol (mmol/L) at 3 months.  2. Mean (SD) change in total /HDL-C ratio at 3 months.  3. Mean (SD) change in body mass index (kg/m^2^) at 3 months.  4. Mean (SD) change in HDL-C (mmol/L) at 3 months.**  **5. Mean (SD) change in LDL-C (mmol/L) at 3 months.  6. Mean (SD) change in systolic BP (mm Hg) at 3 months.  7. Mean (SD) change in diastolic BP (mm Hg) at 3 months. 8. Change in proportion of smokers at 3 months.  9. Mean (SD) change in 8-year coronary risk (%) at 3 months.  10. Mean (SD) change in CV age (years) at 3 months.** | 1. -0.49 (0.99) vs. -0.09 (0.87), *P* < .05 2. -0.6 (1.3) vs. -0.2 (1.2), *P* < .05 3. -0.2 (1.1) vs. -0.3 (1.2), *P* = .31 4. 0.02 (0.17) vs. 0 (0.25), *P* = .55 5. -0.40 (0.87) vs. -0.01 (0.80), *P* < .05 6. -2.0 (14.2) vs. -1.2 (14.1), *P* = .61 7. -0.9 (8.1) vs. 0.1 (9.8), *P* = .99 8. -3 (-1.5%) vs. -2(-2.3%), *P* = .64 9. -1.8% (4.7) vs. -0.3% (5.3), *P* < .01  10. -0.6 (1.8) vs. -0.1 (2.1), *P* < .01 | + | + |
| Rogers 1984[43-45] (USA) | **Prespecified**  **1. Proportion of obesity patients with medical care event at 1 year / 2 years / both years / not done. 1a. Number of diets given or reviewed overall.** 1b. Number of diets given or reviewed for men.  1c. Number of diets given or reviewed for women.   2. **Mean perceived quality of communication score over 1 year adjusted for financial status, chart weight, prior clinic attendance length, and age (high scores better).**  Not prespecified  3. Proportion of times a diagnostic intervention result was recorded for patients with length of hospitalization available (year 1 / year 2). 3a. chest x-ray 3b. electrocardiogram 3c. urine analysis 3d. red blood cells 3e. hemoglobin 3f. hematocrit test (cell pack) 3g. white blood count  3h. blood smear 3i. venereal disease research laboratory 3j. blood urea nitrogen 3k. uric acid 3l. creatinine 3m. fetal bovine serum 3n. PCS (2 hr) 3o. cholesterol 3p. sodium 3q. potassium 3r. chlorides 3s. carbon dioxide 3t. pap smear 3u. all tests | 1a. 16.2% vs. 11.4% / 29.4% vs. 20.3% / 33.8% vs. 20.3% / 20.6% vs. 48.1%, *P* = .01  1b. 15.0% vs. 6.7% / 45.0% vs. 6.7% / 30.0% vs. 46.7% / 10.0% vs. 40.0%, *P* >.05  1c. 16.7% vs. 12.5% / 22.9% vs. 23.4% / 35.4% vs. 14.1% / 25.0% vs. 50.0%, *P* = .02  2. No data reported (figure 2b in article), *P* < .05 in favour of CCDSS.  3a. 82.1% / 84.6% vs. 54.3% / 55.6%  3b. 84.6% / 80.8% vs. 57.1% / 63.0%  3c. 81.6% / 80.8% vs. 48.6% / 66.7%  3d. 71.8% / 73.1% vs. 42.9% / 59.3%  3e. 82.1% / 73.1% vs. 51.4% / 70.4%  3f. 82.1% / 73.1% vs. 57.1% / 66.7%  3g. 87.2% / 73.1% vs. 51.4% / 70.4%  3h. 69.2% / --- vs. 28.6% / ---  3i. 25.6% / 38.5% vs. 20.0% / 22.2%  3j. 87.2% / 88.5% vs. 57.1% / 77.8%  3k. 84.6% / 84.6% vs. 37.1% / 63.0%  3l. 87.2% / 84.6% vs. 42.9% / 63.0%  3m. 84.6% / 88.5% vs. 42.9% / 81.5%  3n. 18.4% / 23.1% vs. 08.6% / 18.5%  3o. 87.2% / 84.6% vs. 45.7% / 63.0%  3p. 82.1% / 88.5% vs. 54.3% / 74.1%  3q. 82.1% / 88.5% vs. 65.7% / 77.8%  3r. 82.1% / 88.5% vs. 54.3% / 74.1%  3s. 82.1% / 88.5% vs. 51.4% / 74.1%  3t. 61.9% / 62.5% vs. 40.0% / 22.7%  3u. 75.3% / 76.2% vs. 45.6% / 61.3% *P* = NR | **Prespecified 1. Mean perceived health status over 1 year adjusted for financial status, chart weight, prior clinic attendance length, and age (high scores better).**   Not clearly prespecified. Data collected by retrospective chart review using a standardized evaluation form. Not clear which data were intended as outcomes for analysis or if post-hoc decision what to analyze. 2. Proportion of deaths by study end. 2a. Obesity patients.  3. Mean adjusted pounds overweight in obesity patients. Adjusted for pounds overweight at baseline, ideal weight, time in cardiac-pulmonary-renal clinics, concomitant diabetes, and total number of other concomitant diseases. Unadjusted data with 95% CIs was also reported in 1982 paper. 3a. Men / women at 10-15 months.  3b. Men / women at 22-24 months.   Not prespecified. 4. Proportion of patients newly diagnosed during study. 4a. Hypertension.  4b. Obesity.  4c. Renal disease.  Note: results for newly diagnosed hypertension and obesity patients generally consistent with those for all patients (4 and 5 above), although at 10-15 months CCDSS patient less overweight (22.1 to 28.2 lbs. vs. 36.7 to 42.6, *P* < .04). | 1. No data reported (figure 2b in article), *P* < .05 in favour of CCDSS.  2a. 1.5% vs. 8.6%, *P* >.05  3a. 45.6 / 52.3 vs. 48.6 / 55.3, *P* = .12  3b. 39.3 / 51.5 vs. 52.2 / 55.8, *P* = .02  4a. 5.8% vs. 4.2%, *P* >.05  4b. 5.4% vs. 8.4%, *P* >.05  4c. 0% vs. 0% | + | + |
| Barnett 1983[17] (USA) | **1. (1 of 2 primary outcomes) number (%) of patients in whom follow-up was attempted or achieved**  1a. 6-12 months **1b. 6-24 months**  **2. (1 of 2 primary outcomes) number (%) of patients for whom a repeat BP measurement was recorded** 2a. 6-12 months **2b. 6-24 months** | 1a. 53 (84%) vs. 13 (25%) (*P* < .01) 1b. 62 (98%) vs. 24 (46%) (*P* < .01)  2a. 31 (49%) vs. 16 (31%) (*P* < .05) 2b. 44 (70%) vs. 27 (52%) (*P* < .05) | **1. (secondary outcome- article states that "blood pressure control in the 2 groups was analyzed, although improved blood pressure control was not an objective of this experiment") degree of BP control** 1a. 6-12 months **1b. 6-24 months** | 1a. 32 (51%) vs. 17 (33%) (*P* < .05) 1b. 44 (70%) vs. 27 (52%) (*P* < .05) | + | + |
| **Screening and management of mental health-related conditions** | | | | | | |
| Ahmad 2009[15] (Canada) | Primary outcomes **1. Opportunity to discuss the possibility of the patient being at risk for intimate partner violence in % (n/N), adjusted RR (95% CI) 2. Detection of intimate partner violence when the patient identified that risk as being present and recent. Intimate partner violence in % (n/N), adjusted RR (95% CI)**  Secondary outcomes 3. physician assessment of patient safety (n/N) 4. provision of appropriate referrals  5. advice for follow-up appointment | 1. 35% (48/139) vs. 24% (34/141); 1.4 (1.1 to 1.9) 2. 18% (25/139) vs. 9% (12/141); 2.0 (0.9 to 4.1) 3. 9/25 vs. 1/12 4. 3/25 vs. 1/12 5. 20/25 vs. 8/12 | ... | ... | + | … |
| Thomas 2004[51] (UK) | **1. % patients satisfied with GP (prespecified) a. at 6 weeks b. at 6 months** | 1a. 75% vs. 72%, *P* = .56 1b. No data reported, NS | **1. GHQ score (lower is better), (95% CI), p-value (primary). a. at 6 weeks b. at 6 months** 2. Mean quality of life score (95% CI), p-value (prespecified) a. at 6 weeks b. at 6 months  3. Recovery rate (%), (95% CI), p-value (prespecified) a. at 6 weeks b. at 6 months | 1a. 14.8 (14.0 to 15.6) vs. 16.0 (15.2 to 16.8), *P* = .04 1b. 14.2 (13.2 to 15.2) vs. 14.5 (13.6 to 15.4), *P* = .61 2a. 5.9 (5.5 to 6.2) vs. 5.8 (5.4 to 6.1), *P* =.73 2b. 6.4 (6.0 to 6.9) vs. 6.2 (5.8 to 6.6), *P* =.52  3a. 38% (33 to 43) vs. 35% (30 to 40), *P* =.38 3b. 35% (30 to 40) vs. 39% (34 to 44), *P* =.20 | 0 | + |
| Schriger 2001[48] (USA) | **Primary Outcome 1. Proportion of patients assigned a psychiatric diagnosis by CCDSS over 18 months (n/N, %, difference, 95% CI) who received:**  1a. Psychiatric diagnosis in emergency department. 1b. Psychiatric consultation or referral in emergency department. **1c. Psychiatric diagnosis, consultation or referral in emergency department.** Prespecified. 2. Proportion of patients with PRIME-MD (computerized interview) or psychiatric diagnosis in emergency department over 18 months. 2a. Any PRIME-MD diagnosis. 2b. 1 PRIME-MD diagnosis. 2c. 2 PRIME-MD diagnoses. 2d. 3 PRIME-MD diagnoses. 2e. >3 PRIME-MD diagnoses.  2f. Any mood diagnosis. 2g. Major depressive diagnosis. 2h. Partial remission of major depressive diagnosis. 2i. Dysthymia. 2j. Minor depressive disorder. 2k. Rule out bipolar disorder. 2l. Any anxiety diagnosis. 2m. Panic disorder.  2n. Generalized anxiety disorder. 2o. Anxiety disorder (not otherwise specified). 2p. Any alcohol abuse/dependence. 2q. Any eating disorder. 2r. Any obsessive-compulsive disorder diagnosis. 2s. Any phobia diagnosis. 3. Items documented in medical encounter over 18 months (% patients). 3a. Psychiatric history. 3b. Notation of alcohol use. 3c. General physical exam. 3d. Eye, ears, nose, throat exam. 3e. Physical exam: CV. 3f. Physical exam: respiratory. 3g. Physical exam: gastrointestinal. 3h. Physical exam: muscular. 3i. Physical exam: neurologic. 3j. Evaluation of orientation and level of consciousness.  3k. Checked 'mood normal' box on chart. 3l. Detailed assessment of affect. 3m. Evaluation of memory, cognition, or reasoning. | 1a. 3/34, 9% vs. 4/45, 9%; 0% (-13 to 14) 1b. 3/34, 9% vs. 3/45, 7%; 2% (-11 to 16) 1c. 6/34, 18% vs. 4/45, 9%; 9% (-8 to 26) 2. N=92 vs. 98 2a. 37% vs. 46% (difference 9%, 95% CI -5 to 23) 2b. 18% vs. 28% 2c. 12% vs. 8% 2d. 2% vs. 7% 2e. 5% vs. 3% 2f. 22% vs. 30% 2g. 16% vs. 16% 2h. 2% vs. 4% 2i. 8% vs. 9% 2j. 4% vs. 8% 2k. 3% vs. 4% 2l. 24% vs. 23% 2m. 10% vs. 3% 2n. 9% vs. 6% 2o. 15% vs. 14% 2p. 9% vs. 15% 2q. 2% vs. 2% 2r. 12% vs. 5% 2s. 3% vs. 4% 3. N=92 vs. 98 3a. 30% vs. 34% 3b. 62% vs. 62% 3c. 94% vs. 97% 3d. 83% vs. 85% 3e. 82% vs. 84% 3f. 86% vs. 84% 3g. 67% vs. 70% 3h. 73% vs. 75% 3i. 67% vs. 67% 3j. 78% vs. 84% 3k. 38% vs. 33% 3l. 5% vs. 7% 3m. 3% vs. 4% | ... | ... | 0 | … |
| Cannon 2000[26] (USA) | **1. Proportion of patients screened for mood disorder over 9 months. (primary) 2. Number, proportion, of major depressive disorder cases with fully documented Diagnostic and Statistical Manual of Mental Disorders (4^th^ edition) diagnostic criteria over 9 months (primary).** | 1. 86.5% vs. 61%, *P* = .01  2. 17/17, 100% vs. 1/18, 5.6%, *P* < .001 | ... | ... | + | … |
| Lewis 1996[37] (UK) | Prespecified: PROQSY group vs. GHQ only group vs. usual care  **1. Mean (SD) number of consultations over 6 months.  1a. Overall.**  1b. Doctor initiated.  1c. Patient initiated.  1d. Physical consults.  1e. Psychological consults.  **2. Proportion of patients (95%CI) with referrals to other professionals (?over 6 months).  2a. To psychological practitioners.**  2b. To other practitioners.  **2c. To other practitioners. PROQSY vs. usual care difference (95% CI).  3. Mean (SD) number of prescriptions (?over 6 months)  3a. Psychotropic drugs.  3b. Non-psychotropic drugs.** | 1a. 3.31 (3.53) vs. 3.33 (3.02) vs. 2.99 (2.91), *P* = .5 1b. 1.30 (1.95) vs. 1.40 (1.98) vs. 1.18 (1.87), *P* = .4 1c. 1.91 (2.18) vs. 1.92 (2.01) vs. 1.79 (1.88), *P* = .7 1d. 2.33 (2.41) vs. 2.39 (2.40) vs. 2.26 (2.26), *P* = .9  1e. 0.79 (2.07) vs. 0.84 (1.92) 0.65 (1.62), *P* = .09  2a. 4.0% (1.8 to 7.4) vs. 5.7% (3.1 to 9.6) vs. 3.5%, (1.5 to 6.8), *P* = .6 2b. 22.5% (17.2 to 28.4) vs. 11.5% (9.1 to 18.3) vs. 15.4% (11.0 to 20.8), *P* = .03 2c. 6.7% (-0.6 to 13.8)  3a. 0.66 (2.33) vs. 0.55 (1.43) vs. 0.44 (1.58), *P* = .6 3b. 2.93 (3.70) vs. 3.43 (4.75) vs. 2.89 (3.32), *P* = .7 | Main outcomes: PROQSY group vs. GHQ only group vs. usual care  1. Mean (95% CI) GHQ score.  1a. At 6weeks.  1b. At 3 months.  1c. At 6 months.  **2. Mean difference (95%CI) in GHQ score PROQSY vs. 2 control groups. 2a. At 6 weeks.  2b. At 3 months.  2c. At 6 months.**  Not prespecified. 3. Proportion of PROQSY-defined cases of mental disorder at 6 weeks, difference (95% CI): PROQSY group vs. usual care.  Note: PROQSY score >11 indicates clinically significant level of distress. | 1a. 25.7 (24.8 to 26.5) vs. 27.2 (26.3 to 28.1) vs. 26.6 (25.7 to 27.5), *P* = .04 in favour of PROQSY 1b. 25.5 (23.8 to 25.8) vs. 27.0 (25.4 to 27.5) vs. 26.4 (25.4 to 27.5), *P* = .07 1c. 25.4 (24.2 to 26.3) vs. 26.8 (25.7 to 27.9) vs. 25.9 (24.2 to 26.6), *P* = .12 2a. 0.92 (0.07 to 1.78)  2b. 0.86 (-0.04 to 1.76) 2c. NS 3. 69.2% vs. 74.5%, 5.3% (-3 to 14) | 0 | 0 |
| Rubenstein 1995[47] (USA) | **1. Mean (SD) and difference (95%CI) for number of clinical problems per patient in medical records during 6 month follow-up that were listed in the visit (prespecified). 2. Mean (SD) and difference (95%CI) for number of functional status interventions per patient with functional status problems during 6 month follow-up (prespecified). 3. Functional status interventions during 6 month follow-up (prespecified).  3a. Total number.  3b. Proportion of interventions recommended in study materials.  4. Physician attitudes toward managing functional status at end of study (prespecified).** | 1. 4.9 (3.4) vs. 4.1 (2.9); 0.8 (0.2 to 1.5), *P* < .01  2. 3.3 (3.7) vs. 2.5 (3.3); 0.8 (0.1 to 1.6), *P* = .05  3a. 231 vs. 95  3b. 81% vs. 71%; 10% (1 to 19), *P* < .02  4. Data not reported. | **1. Mean change (difference, 95% CI) in patient functional status during 6 month follow-up (scale 0-100, 100=highest performance). (predefined) 1a. Basic activities of daily living. 1b. Intermediate activities of daily living. 1c. Mental health. 1d. Social activities. 1e. Work performance.** 2. Mean change adjusted by baseline scores (difference, 95% CI) in social activities scores by age group over 6 month follow-up (unclear if analysis by age groups was preplanned).  2a. <50 years of age. 2b. 50-69 years of age. 2c. >69 years of age.  **3. % patients (difference, 95%CI) identified as having specific impairments during 6 month follow-up (prespecified). 3a. Physical, psychological, or social function impairment.** 3b. Depression or anxiety. 3c. Depression. 3d. Anxiety. 3e. Social problems. 3f. Physical function impairments. | 1a. 0.5 vs. 0.1; 0.44 (-3.2 to 4.1), *P* = .81  1b. 0.9 vs. 1.1; -0.2 (-4.6 to 4.2), *P* = .92  1c. 1.3 vs. -3.2; 4.5 (0.5 to 8.3), *P* = .03  1d. 3.3 vs. -1.5; 4.8 (-0.8 to 10.4), *P* = .09  1e. 0.2 vs. -0.8; 1.0 (-4.4 to 6.6), *P* = .70  2a. 1.13 (n=83) vs. 4.49 (n=79); -3.36 (-9.0 to 2.3), *P* = .24  2b. 1.96 (n=47) vs. -8.31 (n=42); 10.27 (-1.8 to 22.3), *P* = .10  2c. 9.50 (n=40) vs. -10.09 (n=22); 19.59 (1.96 to 36), *P* = .03  Interaction for intervention by age, *P* < .01  3a. 37% vs. 25%; 12% (2 to 2.1), [CIs are not consistent with data although no significant p-value reported for this comparison]  3b. 30% vs. 21%; 9% (1 to 20), *P* < .05  3c. 23% vs. 20%, 3% (-5 to 12), *P* = NS  3d. 13% vs. 4%; 9% (3 to 15), *P* < .01  3e. 17% vs. 10%; 7% (0 to 15), *P* < .10  3f. 6% vs. 5%; 1% (-4 to 5), *P* = NS | … | 0 |
| **Vaccinations** | | | | | | |
| Fiks 2009[1] (USA) | **Primary outcomes over 6 month intervention. 1. Change in rates of captured opportunities for vaccination (visit-level analysis). Pre to post study, difference (95% CI).**  1a. Unadjusted rates.  **1b. Rates adjusted for** **selected covariates.   2. Up-to-date vaccination rates (patient-level analysis). Pre to post study, difference (95% CI).** 2a. Unadjusted rates.  **2b. Rates adjusted for selected covariates.**   Secondary outcomes over 6 months.  3. Difference (95% CI) in proportion of children who had ≥1 vaccine dose (intervention vs. control).   4. Subgroup analyses by site type (4 urban teaching practices or 16 mainly suburban, non-teaching practices) for up-to-date vaccination rates over 6 month study. Pre to post study, difference (95% CI). 4a. Overall effect of practice type on up-to-date vaccination rates.  4b. Urban teaching practices – unadjusted rates.  4c. Non-teaching practices – unadjusted rates.  4d. Urban teaching practices – rates adjusted for selected covariates.  4e. Non-teaching practices – rates adjusted for selected covariates.  5. Secondary analysis limited to visits on days when sites administered ≥ 2 doses of influenza vaccine.  6. Proportion of children with a particular number of visits to the office during the influenza season. Percentage points improvement in intervention practices vs. control practices. 6a. 1 visit 6b. 2 visits 6c. 3 visits 6d. 4 visits  7. Improvement in vaccination rate at intervention sites versus control sites. 7a. Children who had received the influenza vaccine previously 7b. Children who had not received the influenza vaccine previously | 1a. 14.4% to 19.2% vs. 12.3% to 16.1%, 1% (-2.4 to 4.9)  1b. 14.4% to 18.6% vs. 12.7% to 16.3%, 0.3% (-1.9 to 2.5). 2a. 45% to 53% vs. 44.2% to 48.2%, 4.0% (-1.3 to 9.1)  2b. 45.7% to 51% vs. 46% to 47.9%, 3.4% (-1.4 to 9.1)  3. 4.0% (-1.1 to 10.7)  4a. *P* = .23 4b. 46.8% to 59% vs. 47.7% to 53.9%, 6.0% (0.8 to 11.8) 4c. 44% to 49.5% vs. 42.6% to 45.5%, 2.6 (-2.2 to 7.0) 4d. 47.1% to 58.5% vs. 47.8% to 53.8%, 5.4 (1.6 to 9.7) 4e. 44.5% to 46.2% vs. 44.8% to 44.8%, 1.7 (-2.7 to 5.9) 5. data not provided; impact of intervention was similar to impact in #1 above 6. Overall *P* = 0.38 6a. 5% 6b. 5.4% 6c. 9.8% 6d. 7.5% 7. Overall *P* = 0.61 7a. 6.5% 7b. 3.2% | ... | ... | 0 | … |
| Flanagan 1999[32] (USA) | 1. proportion of sessions by all physicians where at least 1 vaccine was ordered (number of sessions with at least 1 order/total sessions) (p-value, RR, 95% CI) (prespecified) **2. number of correct vaccine decisions (main outcome) 2a. Tetanus 2b. Hepatitis 2c. Influenza 2d. Pneumococcal 2e. Measles** 2f. Total 3. number of correct/incorrect tetanus decisions in which ≥2 vaccines were ordered for CCDSS vs. control (not prespecified)  Note: The overall outcome was not evaluated for outcome 2 because no statistical comparison was reported.  Many other results (not prespecified) provided. | 1. 54% (391/726) vs. 67% (169/254) (*P* < .0005, 0.73, 0.60 to 0.87) 2a. 346 vs. 118 (*P* = .77) 2b. 555 vs. 206 (*P* = .14) 2c. 630 vs. 218 (*P* = .75) 2d. 593 vs. 196 (*P* = .12) 2e. 503 vs. 188 (*P* = .17) 2f. 726 vs. 254 (*P* value not provided) 3.88/23 vs. 26/16 (*P* = .04) | ... | ... | 0 | … |
| Chambers 1991[25] (USA) | Prespecified. 4 groups reported: always reminders vs. sometimes reminders (reminder printed vs. no reminder printed) vs. no reminders.  **1. Influenza vaccines given during 2 months of study; n/N (%).**  Subgroup analyses (not clear which, if any, prespecified) 2. Influenza vaccines given during 2 months of study by subgroup (%). 2a. Patient age 0-64 years.  2b. Patient age 65-74 years.  2c. Patient age 75+ years.  2d. Moderate risk level (adapted from Centre for Disease Prevention and Control recommendations).  2e. High risk level (adapted from Centre for Disease Prevention and Control recommendations).  2f. 1 patient visit during study.  2g. 2 patient visits during study.  2h. 3+ patient visits during study.  2i. Primary physician = resident.  2j. Primary physician = attending fellow.   Note: analyses excluded all patients (n=61) of 1 physician in the ‘no reminder’ group who had a high rate of immunization during the study (75%) and in the year before the study (61% compared with <30% for other physicians). | 1. 137/271 (51%) vs. 27/72 (38%) vs. 15/74 (20%) vs. 65/218 (30%), *P* < .001 overall, *P* < .001 for always reminders vs no reminders; Yates-corrected chi-square *P* =.92 for sometimes reminders (printed or not) vs no reminders (latter calculated by research assistant) 2a. 41% vs. 18% vs. 6% vs. 22%, *P* = .001 2b. 48% vs. 43% vs. 33% vs. 31%, *P* = NS  2c. 61% vs. 13% vs. 38% vs. 38%, *P* = .01 2d. 49% vs. 35% vs. 21% vs. 30%, *P* < .001 2e. 55% vs. 45% vs. 19% vs. 28%, *P* = .002 2f. 47% vs. 30% vs. 16% vs. 28%, *P* < .001 2g. 59% vs. 43% vs. 29% vs. 25%, *P* < .001 2h. 45% vs. 55% vs. 14% vs. 42%, *P* = NS 2i. 36% vs. 26% vs. 16% vs. 26%, *P* = NS 2j. 56% vs. 64% vs. 28% vs. 32%, *P* < .001 | ... | ... | + for always reminders  0 for sometimes reminders | … |
| **Other preventive care activities** | | | | | | |
| Sundaram 2009[50] (USA) | **1. Proportion of change in HIV testing rates (primary)**  2. Number (%) of patients tested for HIV, baseline (6 months preintervention) / follow-up (6 months during intervention)  3. Among tested patients, number (%) of tests: baseline (6 months preintervention) / follow-up (6 months during intervention) (secondary)  3a. with documented risk behavior* (including alcohol use only) 3b. with documented risk behavior* (excluding alcohol use only) 3c. patient requested test 3d. reason for test unclear 3e. guideline concordant testing  4. Among untested patients, number (%) of tests: baseline (6 months preintervention) / follow-up (6 months during intervention), % (secondary)  4a. risk assessment done  4b. with documented risk behavior* (including alcohol use) 4c. with documented risk behavior* (excluding alcohol use) 4d. test offered 4e. provider action guideline concordant  * risk behavior defined in article using Centres for Disease Prevention and Control guidelines | 1. 0.29% vs. 0.52%, *P* = .75  2. 98/5484 (1.78%) / 114/6207 (1.84%), *P* = .57 vs. 67/6976 (0.96%) / 106/7375 (1.44%), *P* = .3   3a. 64/98 (65%) / 91/114 (80%) vs. 43/67 (64%) / 81/106 (76%) 3b. 54/98 (55%) / 87/114 (76%) vs. 37/67 (55%) / 70/106 (66%) 3c. 36/98 (37%) / 39/114 (34%) vs. 29/67 (43%) / 44/106 (42%)  3d. 14/98 (14%) / 7/114 (6%) vs. 11/67 (16%) / 11/106 (10%)  3e. 84/98 (86%) / 107/114 (94%) vs. 56/67 (84%) / 94/106 (89%)  4a. 11/154 (7%) / 27/200 (14%) vs. 8/199 (4%) / 8/200 (4%)  4b. 62/154 (40%) / 68/200 (34%) vs. 73/199 (37%) / 71/200 (36%)  4c. 47/154 (31%) / 52/200 (26%) vs. 52/199 (26%) / 53/200 (27%) 4d. 2/154 (1%) / 0/200 (0%) vs. 0/199 (0%) / 2/200 (1%) 4e. 56/154 (36%) / 62/200 (31%) vs. 54/199 (27%) / 67/200 (34%) | ... | ... | 0 | … |
| Lafata 2007[36] (USA) | **The primary pre-determined physician outcome was the  1. unadjusted rate of bone mineral density testing for a 12-month period after the date of first mailing for mailed reminder in combination with physician prompt vs. mailed reminder vs. usual care arm, p-value.** 2. Rate of abnormal findings (hip or spine t-score ≤ -2.0) mailed reminder in combination with physician prompt vs. mailed reminder vs. usual care, p-value (not prespecified). 3. Adjusted bone mineral density testing rates (95% confidence intervals) among patient mailed reminder and physician prompt vs. patient mailed reminder vs. usual care (not prespecified). 3a. Screening at age 65 3b. Screening at age 75 3c. Screening at age 85 4. The secondary pre-determined physician outcome was the dispensing of an osteoporosis medication (treatment rates). (For 5877 receiving bone mineral density test) | 1. 28.9%, vs. 21.4% vs. 10.8%, *P* < .001. 2. 13.7% vs. 17.8% vs. 16.2%, *P* = .10. 3a. 30.3% (27.8 to 32.9) vs. 23.2% (20.6 to 25.9) vs. 17.0% (13.8 to 20.9)  3b. 27.0% (24.7 to 29.4) vs. 18.7% (16.5 to 21.0) vs. 10.1% (8.0 to 12.6)  3c. 23.9% (21.8 to 26.2) vs. 14.8% (13.1 to 16.8) vs. 5.8% (4.5 to 7.3)  4. 3.9% (3.0 to 5.1) vs. 4.0% (2.8 to 5.7) vs. 2.3% (1.6 to 3.3), *P* equals significant for the two active treatments versus usual care. | ... | ... | + | … |
| Zanetti 2003[59] (USA) | **1. Number (proportion) of patients given an intraoperative redose of antibiotics, n (%); adjusted OR (95% CI). (primary outcome)** | 1. 93/137 (68%) vs. 55/136 (40%); 3.31 (1.97 to 5.61), *P* < .001.  Note: 227 vs. 222 randomized; 168 vs. 163 could have reminders activated (i.e., surgery documented as >225 minutes and patient given antibiotics); and 137 vs. 136 were documented as eligible for intraoperative redosing according to guidelines and were included in primary analysis. | **1. Number (proportion) with surgical-site infection. (secondary outcome)** | 1. 5/137 (4%) vs. 8/136 (6%); *P* = .4. | + | 0 |

Abbreviations: BP, blood pressure; CCDSS, computerized clinical decision support system; CHD, coronary heart disease; CI, confidence interval; CV(D), cardiovascular disease; FOBT, fecal occult blood test; GHQ, general health questionnaire; GP, general practitioner; HDL-C, high-density lipoprotein cholesterol; HIV, human immunodeficiency virus; HMO, health maintenance organization; ITT, intention to treat; LDL-C, low-density lipoprotein cholesterol; LLD, lipid-lowering drug; NR, not reported; NS, not significant; OR, odds ratio; PRIME-MD, Primary Care Evaluation of Mental Disorders; RR, relative risk; SD, standard deviation.

^a^Ellipses (…) indicate item was not assessed or could not be evaluated. Outcomes in bold font were assessed for effect.

^b^Outcomes are evaluated for effect as positive (+) or negative (−) for CCDSS, or no effect (0), based on the following hierarchy. An effect is defined as ≥ 50% of relevant outcomes showing a statistically significant difference (2*P* < .05):

1. If a single primary outcome is reported, *in which all components are applicable*, this is the only outcome evaluated.
2. If >1 primary outcome is reported, the ≥50% rule applies and only the primary outcomes are evaluated.
3. If no primary outcomes are reported (or only some of the primary outcome components are relevant) but overall analyses are provided, the overall analyses are evaluated as primary outcomes. Subgroup analyses are not considered.
4. If no primary outcomes or overall analyses are reported, or only some components of the primary outcome are relevant for the application, any reported prespecified outcomes are evaluated.
5. If no clearly prespecified outcomes are reported, any available outcomes are considered.
6. If statistical comparisons are not reported, ‘effect’ is designated as not evaluated (…).
