# Supplementary material for: Computerized clinical decision support systems for primary preventive care: A decision-maker-researcher partnership systematic review of effects on process of care and patient outcomes
Source: Implement Sci. 2011 Aug 3;6:87. doi: 10.1186/1748-5908-6-87 (PMC3173370; doi:10.1186/1748-5908-6-87)
Supplement: Additional file 5 — Costs and CCDSS process-related outcomes for trials of primary preventive care. Cost and CCDSS process-related outcomes for the included studies. [file 1748-5908-6-87-S5.DOCX]

**Additional file 5, Table S5. Costs and CCDSS process-related outcomes for trials of primary preventive care**

| **Study**  **(country)** | **Method ccore** | **CCDSS adverse effects** | **Costs** ^b^ | **Group comparison for CCDSS workflow** | **Practitioner satisfaction with CCDSS system** |
| --- | --- | --- | --- | --- | --- |
| **Cancer screening** | | | | | |
| Sequist, 2009[49]  (USA) | 9 | … | ... | ... | 47% of practitioners reported that electronic reminders were "somewhat effective" while 9% reported that they were "very effective" at increasing colorectal screening. Physicians reported that 50% (median) of reminders accurately reflected patients' screening status (IQR 30% to 80%). |
| Emery, 2007[30]  (UK) | 10 | … | ... | ... | Practitioner attitudes increased from pre-training to 12 months post-training:  a. Confidence in managing familial cancer risk, *P* < .001  b. Using software is simple, *P* < .001  c. Using software is cost-effective, *P* = .02  d. Using software is beneficial, *P* = .02  e. Using software is easy, *P* < .001  f. Using software is desirable, *P* = .04 |
| Wilson, 2005[57, 58]  (Scotland)  (UK) | 6 | … | Cost for the software development; total average cost per compact disc (£), marginal cost (£)  1. Staff – 59.38, 2.06  2. Consumables 1.06, 1.06  3. Equipment 1.38, not stated  4. Rooms 9.87, not stated  Total cost 71.69, 3.12  Cost for each GP attending postgraduate education session (£), marginal cost (£)  5. Staff – software team 21.20, not stated  6. Staff – GP 71.12, 72.12  7. Consumables 1.09, 1.09  8. Equipment 0.16, not stated  9. Room 7.11, not stated  10. GP travel costs 4.39, 4.39  11. Total cost 106.07, 77.60 | ... | ... |
| McPhee, 1991[40]  (USA) | 7 | … | ... | ... | Author reported data were not formally collected; however, >90% were satisfied with the system on periodic follow-up visits. |
| **Multiple preventive care activities** | | | | |  |
| Apkon, 2005[16]  (USA) | 5 | … | 1. Mean time to coordinate Coupler session = 18 minutes  2. Median (IQR) resource consumption over 60 days ($): CCDSS vs. control  a. Ambulatory visits. 307 (153 to 613) vs. 292 (146 to 541), *P* = .17  b. Lab testing. 43 (0 to 144) vs. 31 (0 to 139), *P* = .04  c. Diagnostic imaging. 31 (0 to 148) vs. 29 (0 to 127), *P* = .26  d. Pharmacy use. 203 (68 to 495) vs. 164 (50 to 453), *P* = .03  e. Total. 789 (375 to 1654) vs. 698 (340 to 1530), *P* = .05 | ... | Provider satisfaction at 60 days  • 75% agreed/strongly agreed that Couplers provided high-quality information  • 83% disagreed/strongly disagreed that time to use Couplers was acceptable  • 70% disagreed that Couplers were beneficial for medical decision-making  • 61% disagreed that Couplers improved provider-patient interactions  • 70% disagreed that Couplers provided overall benefits to patients. |
| Frame, 1994[33]  (USA) | 6 | … | Provider time same for both groups. Cost of maintaining system, generating reminders, and mailing patient reminders, 78c/patient/year, with 67% of cost for patient reminders.  Estimated costs/1,000 patient/provider reminders ($):  a. Staff ($9.85/hour). 162.53/182.23  b. Materials. 92.50/52.50  c. Postage ($0.29/envelope). 290.00/0  d. Total. 545.03/234.73  This was not offset by increased billings for Intervention vs. control (total $).  Billings:  a. Preintervention. 54,834 vs. 48,150  b. Intervention year 1. 58, 201 vs. 55,823  c. Intervention year 2. 57,604 vs. 57,014 | ... | ... |
| Rosser, 1991[46]  (Canada) | 6 | … | No cost data provided, although author reported that physician reminder was the most cost-effective method of improving preventive services; letter reminder was next, and telephone reminders the least cost-effective. "Cost-effectiveness was calculated by determining the cost of each procedure completed in excess of the number completed in the control group." Author has indicated he believes cost is <$5 per extra procedure completed. | ... | ... |
| **Screening and management of CV risk factors** | | | | |  |
| Bertoni, 2009[18, 19]  (USA) | 9 | Patients had a greater risk for overtreatment than of undertreatment because all patients were screened including low-risk patients who would not normally be screened. | ... | ... | ... |
| Unrod, 2007[54, 55]  (USA) | 8 | … | Costs associated with implementing the expert system intervention vs. control  1. Total workstation costs (excluding PCP) $2,382.60 vs. $0  2. PCP training cost $131.60 vs. $0  3. Total workstation and training costs $2,514.20 vs. $0  4. Total upfront costs/computer lifetime smokers $8.82 vs. $0  5. Office administrator “asking” cost per smoker $4.02 vs. $0  6. Office administrator assistance time per smoker $0.42 vs. $0  7. PCP report review per smoker (including brochure) for initial visit $14.48 vs. $3.08  8. PCP report review per smoker (including brochure) for follow-up visit $7.26 vs. $5.06  9. PCP report review per smoker (including brochure) total $21.74 vs. $8.14  10. Total practice costs per smoker $42.10 vs. $8.14  11. Adjuvant therapy costs per smoker $47.08 vs. $40.21  12. Total cessation costs per smoker by state of readiness to quit, pre-preparation $85.66 vs. $38.45  13. Total cessation costs per smoker by state of readiness to quit, preparation $94.17 vs. $61.73  14. Total cessation costs per smoker by state of readiness to quit, total $89.18 vs. 48.35  15. Total cessation costs per practice excluding adjuvant therapy $5,742.44 vs. $1,110.30  16. Total cessation costs $12,164.15 vs. $6,594.94  Physicians in the intervention arm viewed the expert system report 95% of the time for an average of 2.5 minutes and spent 4.2 minutes discussing the five “A’s | ... | ... |
| Cobos, 2005[27]  (Spain) | 10 | ... | Direct costs = sum of costs of physician visits, laboratory analyses, and LLDs prescribed during the study. For each patient, visit and laboratory costs were estimated by frequency x unit cost (physician visit €12, lipid €9.46, alanine aminotransferase and aspartate aminotransferase €2 each, creatine kinase €1). All costs in Euros (€).  1. LLD treatment costs at one year. 125,569 vs. 214,683  2. LLD total costs at one year. 170,061 vs. 264,658  3. Adjusted means for treatment costs per patient; difference (95% CI); savings %. 178 vs. 237; 59 (34 to 83, *P* < .00001); 24.9%.  4. Adjusted means for total costs per patient; difference (95% CI); savings %. 223 vs. 283; 60 (33 to 86), *P* = .001; 20.8% | ... | ... |
| **Screening and management of mental health-related conditions** | | | | |  |
| Ahmad, 2009[15]  (Canada) | 8 | … | … | … | “In interviews after the trial, physicians commented on the tool's usefulness for identifying psychosocial issues, particularly for annual visits and preventive care.” |
| **Other preventive care activities** | | | | |  |
| Sundaram, 2009[49]  (USA) | 7 | … | … | … | 78% of the intervention providers who received feedback felt it had an effect on their HIV test ordering practices. |
| Zanetti, 2003[59]  (USA) | 8 | 1-Inappropriate activation of the system, n, %. 4/449 procedures (1%).  2- Unnecessary intraoperative redosing, n=1. | … | ... | ... |

Abbreviations: CCDSS, computerized clinical decision support system; CI, confidence interval; GP, general practitioner; HIV, human immunodeficiency virus; IQR, interquartile range; LLD, lipid-lowering drug; PCP, primary care provider.

^a^ Ellipses (…) indicate outcome was not assessed.

^b^ Costs include workflow measures such as time to process alerts if these are not directly compared between groups.
